# Cytotoxic and mutagenic properties of *O*^4^-alkylthymidine lesions in *Escherichia coli* cells

**DOI:** 10.1093/nar/gkv941

**Published:** 2015-09-22

**Authors:** Pengcheng Wang, Nicholas J. Amato, Qianqian Zhai, Yinsheng Wang

**Affiliations:** 1 Environmental Toxicology Graduate Program,University of California, Riverside, CA 92521-0403, USA; 2 Department of Chemistry, University of California, Riverside, CA 92521-0403, USA

## Abstract

Due to the abundant presence of alkylating agents in living cells and the environment, DNA alkylation is generally unavoidable. Among the alkylated DNA lesions, *O*^4^-alkylthymidine (*O*^4^-alkyldT) are known to be highly mutagenic and persistent in mammalian tissues. Not much is known about how the structures of the alkyl group affect the repair and replicative bypass of the *O*^4^-alkyldT lesions, or how the latter process is modulated by translesion synthesis polymerases. Herein, we synthesized oligodeoxyribonucleotides harboring eight site-specifically inserted *O*^4^-alkyldT lesions and examined their impact on DNA replication in *Escherichia coli* cells. We showed that the replication past all the *O*^4^-alkyldT lesions except (*S*)- and (*R*)-*s*BudT was highly efficient, and these lesions directed very high frequencies of dGMP misincorporation in *E. coli* cells. While SOS-induced DNA polymerases play redundant roles in bypassing most of the *O*^4^-alkyldT lesions, the bypass of (*S*)- and (*R*)-*s*BudT necessitated Pol V. Moreover, Ada was not involved in the repair of any *O*^4^-alkyldT lesions, Ogt was able to repair *O*^4^-MedT and, to a lesser extent, *O*^4^-EtdT and *O*^4^-*n*PrdT, but not other *O*^4^-alkyldT lesions. Together, our study provided important new knowledge about the repair of the *O*^4^-alkyldT lesions and their recognition by the *E. coli* replication machinery.

## INTRODUCTION

The genomic integrity is constantly challenged by endogenous metabolism and environmental exposure, leading to a diverse array of damage products in DNA (1,2). Alkylation of DNA is generally unavoidable owing to the ubiquitous presence of alkylating agents in the environment and in living cells, generating adducts at multiple sites on nucleobases as well as the phosphate backbone (3–6). Depending on the nature of the alkylating agents involved, the size of alkyl groups adducted to DNA varies from a simple methyl group to complex alkyl functionalities (6,7). Despite the cytotoxic, teratogenic and carcinogenic effects, alkylating agents also constitute a major class of cancer chemotherapeutic drugs (8).

Among all DNA alkylation adducts, *O*^6^-alkyl-2′-deoxyguanosine (*O*^6^-alkyldG) and *O*^4^-alkylthymidine (*O*^4^-alkyldT) are known to be highly mutagenic (6,9–11). Although *O*^6^-alkyldG is induced more efficiently than *O*^4^-alkyldT (6) and can be detected both *in vivo* and *in vitro* (12), *O*^4^-alkyldT was found to accumulate at higher levels than *O*^6^-alkyldG in cellular and tissue DNA (13–16), suggesting that *O*^4^-alkyldT may be a more or equally important DNA alkylation adduct. Thus, it is important to understand how the *O*^4^-alkyldT lesions perturb the efficiency and accuracy of DNA replication, and how they are repaired.

**Scheme 1.**
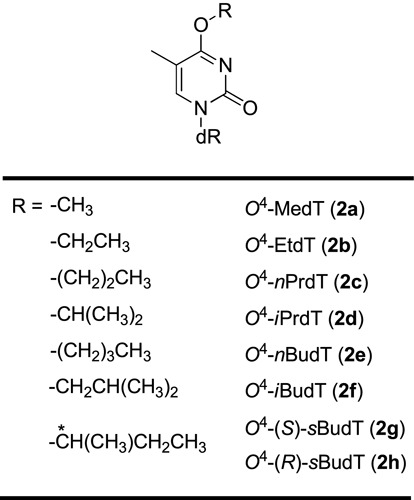
Structures of the *O*^4^-alkyldT lesions examined in the present study.

Several shuttle vector studies with the use of site-specifically incorporated DNA lesions have been conducted for assessing how *O*^4^-alkyldT lesions compromise DNA replication in *Escherichia coli* and mammalian cells. In this vein, it was observed that *O*^4^-methyl- and *O*^4^-ethyl-dT (*O*^4^-MedT and *O*^4^-EtdT, Scheme 1) could direct high frequencies of dGMP misincorporation in *E. coli* cells (17–20). Likewise, *O*^4^-MedT, *O*^4^-EtdT and *O*^4^-*n*-propryl-dT (*O*^4^-*n*PrdT, Scheme 1) were found to be highly mutagenic during replication in mammalian cells (21–24), with the former two inducing T→C transition mutations at higher frequencies (∼20%) than *O*^4^-*n*PrdT (∼12%) (23). The relatively low mutation frequency of *O*^4^-*n*PrdT was attributed to the more efficient repair of this lesion, presumably by the nucleotide excision repair pathway (23). The miscoding properties of these lesions revealed from the *in* *vivo* replication experiments are in keeping with the findings made from *in* *vitro* replication studies, where *O*^4^-MedT, *O*^4^-EtdT and *O*^4^-*iso*-propyl-dT (*O*^4^-*i*PrdT, Scheme 1) were shown to direct preferential misincorporation of dGMP by purified DNA polymerases (25–27). However, very little is known about the roles of translesion synthesis DNA polymerases in bypassing these lesions in cells (20).

Previous studies showed that *O*^6^-alkylguanine-DNA alkyltransferase (AGT) was relatively inefficient in repairing the *O*^4^-alkyldT lesions (28–32). *In vitro* biochemical assays revealed that purified *E. coli* Ada repairs *O*^4^-MedT 10 000 times slower than *O*^6^-MedG, and Ogt repairs *O*^4^-MedT at a ∼84-fold higher efficiency than Ada (29,30). The lack of involvement of Ada in repairing *O*^4^-MedT was also manifested by the observation that the mutation frequency of *O*^4^-MedT, when situated in a single-stranded plasmid, was not altered by the depletion of this repair protein in *E. coli* cells (17). In addition, a recent study showed that the T:A→C:G transition mutations induced by ethylating agents and propylating agents in *E. coli* cells could be efficiently reduced by Ogt, suggesting the involvement of Ogt in repairing *O*^4^-EtdT and *O*^4^-*n*PrdT (32). Considering that these alkylating agents could also induce alkylation at adenine and at other nucleophilic sites (i.e., *O*^2^ and *N*3) on thymine, it is not clear whether the reduction in T:A→C:G transition mutations is primarily attributed to the repair of *O*^4^-alkyldT lesions. Thus, it is important to assess systematically the AGT-mediated repair of *O*^4^-alkyldT lesions and the modulation of this repair by the structure of the alkyl group.

Although site-specific mutagenesis studies have been carried out for several *O*^4^-alkyldT lesions, the studies were conducted with the use of somewhat disparate shuttle vector systems relying on colony picking/counting and sequencing to estimate the degrees to which these lesions impede DNA replication and induce mutations, respectively. In the present study, we set out to assess comprehensively, by using a highly quantitative and accurate shuttle vector-based assay (33,34), how the size (from methyl to butyl), branching (*i*Pr, *i*Bu and *s*Bu versus their straight chain counterparts) and stereochemsitry (*R* versus *S* diastereomers of *s*Bu) of the alkyl group incorporated to the major-groove *O*^4^ position of thymine affect the fidelity and efficiency of DNA replication, and how replication past these lesions is affected by AGT proteins and SOS-induced DNA polymerases.

## MATERIALS AND METHODS

### Material

All chemicals, unless otherwise specified, were purchased from Sigma-Aldrich (St Louis, MO, USA) or EMD Millipore (Billerica, MA, USA). 1,1,1,3,3,3-Hexafluoro-2-propanol (HFIP) was obtained from Oakwood Products Inc. (West Columbia, SC, USA). Common reagents for solid-phase DNA synthesis were obtained from Glen Research Co. (Sterling, VA, USA) and unmodified oligodeoxyribonucleotides (ODNs) were from Integrated DNA Technologies (Coralville, IA, USA). [γ-^32^P]ATP was obtained from Perkin Elmer (Piscataway, NJ, USA). Shrimp alkaline phosphatase was purchased from USB Corporation (Cleveland, OH, USA) and all other enzymes were obtained from New England Biolabs (Ipswich, MA, USA).

M13mp7(L2), wild-type AB1157 and C215 *E. coli* strains, and Ada- and Ogt-deficient *E. coli* strains (as FC215 derivatives), including C216 (Δ*ogt::kan*), C217 (Δ*ada* Δ*alkB::cam*) and C218 (Δ*ogt::kan* Δ*ada* Δ*alkB::cam*), were kindly provided by Prof. John M. Essigmann (35). Polymerase-deficient AB1157 strains [Δ*pol B1*::spec (Pol II-deficient), Δ*dinB* (Pol IV-deficient), Δ*umuC*::kan (Pol V-deficient) and Δ*pol B1*::spec*ΔdinB* Δ*umuC*::kan(Pol II, Pol IV, Pol V-triple knockout)] were generously provided by Prof. Graham C. Walker (36).

### Chemical syntheses

**Scheme 2.**
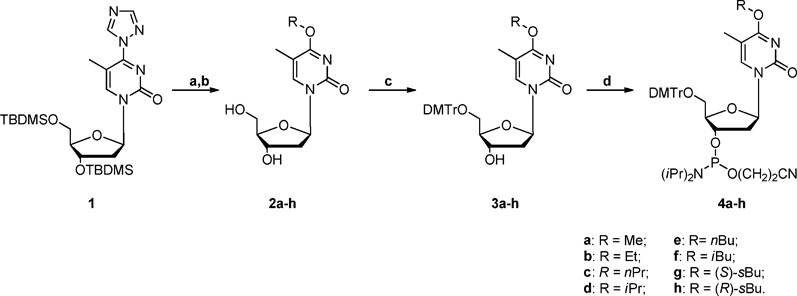
Syntheses of phosphoramidite building blocks of *O*^4^-alkylthymidine^α^. ^α^Reagents and conditions: (a) ROH, DBU, r.t., 10 h; (b) TBAF, THF, r.t., 1 h; (c) DMTr-Cl, DMAP, pyridine, r.t., 10 h; (d) 2-cyanoethyl-*N*,*N*-diisopropyl chlorophosphoramidite, DIEA, CH_2_Cl_2_, 1 h.

Eight *O*^4^-alkyldT derivatives were synthesized (Scheme 1). The synthetic route for the phosphoramidite building blocks of the *O*^4^-alkyldT lesions was adapted from previously published procedures (Scheme 2) (37).

### General procedures for the syntheses of *O*^4^-alkylthymidine

Compound **1** (300 mg, 0.58 mmol), prepared at an overall yield of 81% according to the published procedures (37), was dissolved in 6 ml of anhydrous acetonitrile at 0°C, to which solution were added 1,8-diazabicyclo[5.4.0]undec-7-ene (DBU, 0.17 ml, 1.16 mmol) and the corresponding alcohol (1 ml). After 30 min, the resulting mixture was allowed to warm to r.t. and the solution was stirred overnight. The solution was neutralized by addition of 0.5 M aqueous solution of KH_2_PO_4_ (pH 6.5) and the product was extracted into chloroform (40 ml). The organic layer was then washed with brine (3 × 20 ml) and dried over anhydrous Na_2_SO_4_. The filtrate was concentrated and the residue was dissolved in 4 ml of anhydrous tetrahydrofuran (THF), to which 0.26 ml of tetrabutylammonium fluoride (TBAF) in THF (1.0 M) was added. The resulting mixture was stirred for another 2 h and concentrated. The residue was purified by silica gel column chromatography with a step gradient of methanol (0–7%) in methylene chloride to afford the desired product **2a-h**. In this context, the stereospecific syntheses of *O*^4^-(*S*)-*s*BudT and *O*^4^-(*R*)-*s*BudT were accomplished by employing the commercially available (*S*)-*s*BuOH and (*R*)-*s*BuOH, respectively.

### General procedures for DMTr protection

To 100 mg of compound **2a-h**, which was dried three times by evaporation with pyridine and dissolved in anhydrous pyridine (10 ml) on an ice bath, were added 4-dimethylaminopyridine (DMAP, 0.5% mol) and 4,4′-dimethoxytrityl chloride (DMTr-Cl, 1.2 eq.). The resulting solution was stirred at room temperature for 10 h. The reaction was then quenched with methanol (0.5 ml), and the solvent was removed *in vacuo*. The residue was purified by silica gel column chromatography with ethyl acetate as the mobile phase to yield the desired product **3a-h**.

### General procedures for phosphoramidite synthesis

To a round bottom flask, which was stirred in an ice bath and contained a solution of compounds **3a-h** (60 mg) in anhydrous methylene chloride (3.0 ml), were added *N*,*N*-diisopropylethylamine (DIEA, 2.2 eq.) and 2-cyanoethyl-*N*,*N*-diisopropyl chlorophosphoramidite (1.2 eq.). The mixture was then stirred at room temperature for 1 h under an argon atmosphere. The reaction was quenched by cooling the mixture in an ice bath followed by slow addition of methanol (0.20 ml). The solution was quickly diluted with ethyl acetate (8.0 ml). The organic layer was washed sequentially with saturated NaHCO_3_ (4.0 ml) and brine (4.0 ml), and dried over anhydrous Na_2_SO_4_. The solvent was evaporated under reduced pressure to yield **4a-h** in a foam that was used directly for ODN synthesis.

The reaction yields and spectroscopic characterizations of the above-synthesized products are provided in the online Supplementary Materials. The nuclear magnetic resonance spectra for these compounds are shown in Supplementary Figures S1–S18.

### ODN synthesis

The 12-mer lesion-containing ODNs 5′-ATGGCGXGCTAT-3′ (‘X’ represents the *O*^4^-alkyldT lesions) were synthesized on a Beckman Oligo 1000S DNA synthesizer (Fullerton, CA) at 1 μmol scale. The synthesized phosphoramidite building block was dissolved in anhydrous acetonitrile at a concentration of 0.067 M. Commercially available phosphoramidite building blocks (ultramild) were employed for the incorporation of the unmodified nucleotides (Glen Research Co., Sterling, VA, USA) following the standard ODN assembly protocol. The ODNs were cleaved from the controlled pore glass (CPG) support and deprotected with concentrated ammonium hydroxide at r.t. for 1 h. The solvents were evaporated, and the residues were redissolved in water and purified by high-performance liquid chromatography (HPLC).

### HPLC

HPLC separations were conducted on an Agilent 1100 HPLC system with a Kinetex XB-C18 column (4.60 × 150 mm, 5 μm in particle size and 100 Å in pore size; Phenomenex Inc., Torrance, CA, USA). For the purification of ODNs, a triethylammonium acetate buffer (50 mM, pH 6.8, Solution A) and a mixture of solution A and acetonitrile (70/30, v/v, Solution B) were employed as mobile phases. The flow rate was 0.8 ml/min and the gradient profile was 5–25% B in 5 min followed by 25–55% B in 60 min. The HPLC traces for the purifications of the 12-mer lesion-containing ODNs are shown in Supplementary Figure S19 and the electrospray ionization-mass spectrometry (ESI-MS) and tandem MS (MS*/*MS) of the purified lesion-containing ODNs are displayed in Supplementary Figures S20–S26.

### Preparation of the lesion-carrying 22-mer ODNs

The 12-mer *O*^4^-alkyldT-containing ODNs were 5′-phosphorylated and ligated individually with a 10-mer ODN (5′-AGTGGAAGAC-3′) in the presence of a template in the ligation buffer with T4 DNA ligase and ATP at 16°C for 8 h. The resulting 22-mer ODNs were purified by denaturing PAGE.

### Construction of single-stranded lesion-containing and lesion-free competitor M13 genomes

The lesion-containing and lesion-free M13mp7(L2) genomes were prepared following the previously reported procedures (Supplementary Figure S27) (38). First, 20 pmol of single-stranded M13 genome was digested with 40 U EcoRI at 23°C for 8 h to linearize the vector. The resulting linearized vector was then mixed with two scaffolds, 5′-CTTCCACTCACTGAATCATGGTCATAGCTTTC-3′ and 5′-AAAACGACGGCCAGTGAATTATAGC-3′ (25 pmol), each spanning one end of the linearized vector. To the mixture a 30 pmol of the 5′-phosphorylated 22-mer *O*^4^-alkyldT-bearing ODN or the competitor ODN (25-mer, 5′-GCAGGATGTCATGGCGATAAGCTAT-3′) was subsequently added, and the DNA was annealed. The resulting mixture was treated with T4 DNA ligase at 16°C for 8 h, followed by incubation with T4 DNA polymerase (22.5 U) at 37°C for 4 h to degrade the excess scaffolds and the unligated vector. The lesion-containing and the lesion-free M13 genomes were purified from the solution by using Cycle Pure Kit (Omega). The constructed lesion-containing genomes were normalized against the lesion-free competitor genome following published procedures (38).

### Transfection of control, lesion-containing and competitor M13 genomes into *E. coli* cells

The control lesion-free M13 genome was mixed with competitor genome at a molar ratio of 1:1, the M13 genomes containing the *O*^4^-alkyldT lesions with the alkyl group being Me, Et, *n*Pr and *n*Bu were mixed individually with the competitor genome at a molar ratio of 2:1, and those with the alkyl group being *i*Pr, *i*Bu, (*S*)-*s*Bu and (*R*)-*s*Bu were mixed separately with the competitor genome at a molar ratio of 5:1 (25 fmol each of competitor genome was used). The mixtures were transfected into SOS-induced, electrocompetent wild-type AB1157 *E. coli* cells and the isogenic *E. coli* cells that are deficient in Pol II, Pol IV, Pol V or all three polymerases, as well as wild-type *E. coli* cells and isogenic strains that are deficient in Ogt, Ada or both, following the previously published procedures (38). The SOS induction was achieved by irradiating the *E. coli* cells with 254 nm light at a dose of 45 J/m^2^ (36). The *E. coli* cells were subsequently grown in lysogeny broth (LB) medium at 37°C for 6 h. The phage was recovered from the supernatant by centrifugation at 13 000 r.p.m. for 5 min and further amplified in SCS110 *E. coli* cells to increase the progeny/lesion-genome ratio. The amplified phage was finally purified using the QIAprep Spin M13 kit (Qiagen) to obtain the single-stranded M13 DNA template for polymerase chain reaction (PCR) amplification.

### Quantification of bypass efficiencies and mutation frequencies

We employed a modified version of the competitive replication and adduct bypass (CRAB) assay to assess the bypass efficiencies and mutation frequencies of the *O*^4^-alkyldT lesions upon replication in *E. coli* cells (Supplementary Figure S28) (33,34,38). The PCR amplification was carried out with the use of Phusion high-fidelity DNA polymerase for the sequence region of interest in the single-stranded M13 DNA template. The primers were 5′-YCAGCTATGACCATGATTCAGTGAGTGGA-3′ and 5′-YTCGGTGCGGGCCTCTTCGCTATTAC-3′ (‘Y’ represents a 5′-amino modifier, i.e., H_2_N(CH_2_)_6_-, added to the 5′ phosphate group of the ODNs). The amplification cycles were 30, with each cycle consisting of 10 s at 98°C, 30 s at 65°C and 15 s at 72°C, along with a final extension at 72°C for 5 min. The PCR products were purified by using Cycle Pure Kit (Omega). For the determination of bypass efficiency, a portion of the above PCR products was digested with 10 U BbsI restriction endonuclease and 1 U shrimp alkaline phosphatase in 10 μl New England Biolabs (NEB) CutSmart buffer at 37°C for 30 min, followed by heating at 80°C for 20 min to deactivate the phosphatase. To the above mixture were subsequently added 5 mM DTT, 1 μM ATP (premixed with 1.66 pmol [γ-^32^P]ATP) and 10 U T4 polynucleotide kinase in a 15 μl solution and the mixture was incubated at 37°C for 30 min, followed by heating at 65°C for 20 min to deactivate the T4 polynucleotide kinase. To the resulting solution was added 10 U MluCI restriction endonuclease, and the mixture was incubated at 37°C for 30 min, followed by quenching with 15 μl formamide gel loading buffer containing xylene cyanol FF and bromophenol blue dyes. The mixture was separated by 30% native polyacrylamide gel (acrylamide:bis-acrylamide = 19:1). The DNA bands were quantified using a Typhoon 9410 variable mode Imager.

The above restriction digestion of the PCR products gave rise to a short duplex, d(p*GGCGMGCTAT)/d(AATTATAGCN), where ‘M’ represents the nucleobase incorporated at the original lesion site during DNA replication *in vivo*, ‘N’ is the complementary base of ‘M’ in the opposite strand and ‘p*’ designates the [5′-^32^P]-labeled phosphate (Supplementary Figure S28). The bypass efficiency was determined by using the following formula:

Bypass efficiency (%) = (lesion signal/competitor signal)/(non-lesion control signal/competitor signal) × 100%.

### Identification of mutagenic products by LC-MS/MS

The PCR products were digested with 50 U each of BbsI and MluCI, along with 20 U shrimp alkaline phosphatase in 250 μl NEB CutSmart buffer at 37°C for 2 h, followed by deactivation of enzymes at 80°C for 20 min. The resulting solution was extracted once with phenol/chloroform/isoamyl alcohol (25:24:1, v/v). The aqueous layer was subsequently dried in a Speed-vac, desalted with HPLC and dissolved in 20 μl water. A 10-μl aliquot was injected for LC-MS/MS analysis and an Agilent Zorbax SB-C18 column (0.5 × 250 mm, 5 μm in particle size) was used. The gradient for LC-MS/MS analysis was 5 min of 5–20% methanol followed by 35 min of 20–50% methanol in 400 mM HFIP (pH was adjusted to 7.0 with triethylamine). The temperature for the ion-transport tube was maintained at 300°C. The LTQ linear ion trap mass spectrometer (Thermo Electron, San Jose, CA, USA) was set up for monitoring the fragmentation of the [M-3H]^3−^ ions of the 10-mer d(GGCGMGCTAT) and d(AATTATAGCN), with ‘M’ and ‘N’ being ‘A’, ‘T’, ‘C’ or ‘G’. The fragment ions detected in the MS/MS were manually assigned.

## RESULTS

The major objectives of this study were to investigate how the *O*^4^-alkyldT lesions with varying structures of the alkyl group compromise DNA replication and to define the respective roles of SOS-induced DNA polymerases and *O*^6^-alkylguanine-DNA alkyltransferases in bypassing and repairing these lesions in *E. coli* cells. To this end, we first synthesized lesion-carrying ODNs with a site-specifically incorporated *O*^4^-alkyldT (Scheme 1) and characterized these ODNs by ESI-MS and MS*/*MS analyses (Supplementary Figures S20–S26). The LC-MS results revealed no detectable level of degradation of *O*^4^-alkyldT to dT in these modified ODNs.

**Figure 1.**
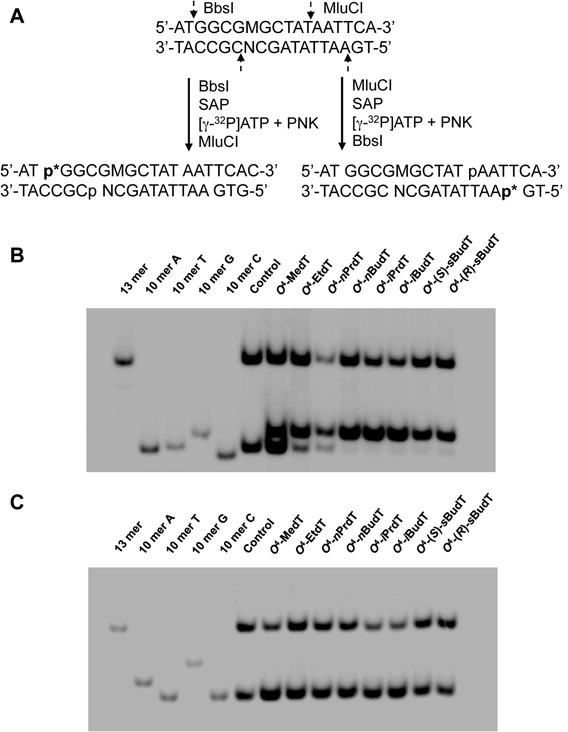
Native PAGE (30%) for monitoring the bypass efficiencies and mutation frequencies of *O*^4^-alkyldT in SOS-induced wild-type (WT) AB1157 *Escherichia coli* cells. (**A**) Sequential restriction enzyme digestion for the selective labeling of the strand initially bearing the lesion or the complementary strand. ‘SAP’ and ‘PNK’ designate shrimp alkaline phosphatase and T4 polynucleotide kinase, respectively. (**B**) Gel image showing the 13-mer and 10-mer products released from the bottom-strand (opposite to lesion-containing strand) of the PCR products of the progeny of the competitor genome and the control or lesion-carrying genome, where 10mer A, 10mer C, 10mer G and 10mer T represent the [5′-^32^P]-labeled standard ODNs 5′-AATTATAGCN-3′, with ‘N’ being A, C, G and T, respectively. (**C**) Gel image showing the 13-mer and 10-mer products released from the top-strand (lesion-containing strand) of the PCR products of the progeny of the competitor genome and the control or lesion-carrying genome, where 10mer A, 10mer C, 10mer G and 10mer T represent the [5′-^32^P]-labeled standard ODNs 5′-GGCGMGCTAT-3′, with ‘M’ being A, C, G and T, respectively.

To assess the bypass efficiencies and mutation frequencies of the *O*^4^-alkyldT lesions, we ligated the aforementioned lesion-containing ODNs into single-stranded M13 genome and performed a modified version of the CRAB and restriction endonuclease and post-labeling (REAP) assays to examine how these lesions inhibit DNA replication and induce mutations in *E. coli* cells (Supplementary Figure S28). We employed two restriction enzymes, i.e. MluCI and BbsI, to digest the PCR products of the progeny genome, affording 10mer ODN fragment(s) from the lesion-containing or lesion-free genome and a 13mer fragment from the competitor genome (Figure 1). The released ODNs were subjected to LC-MS/MS and native PAGE analyses to identify the replication products (Supplementary Figures S29–S39), as described elsewhere (20,39). As illustrated in Figure 1, by switching the order of the two restriction enzyme digestion, we can selectively incorporate a ^32^P-labeled phosphate to the 5′ ends of lesion-situated strand, i.e. d(p*GGCGMGCTAT) or its complementary strand, i.e. d(p*AATTATAGCN). When BbsI was first added, we could resolve, with 30% native PAGE, the [5′-^32^P]-labeled d(p*GGCGTGCTAT) (non-mutagenic product, 10mer-T) from the products carrying a T→A or T→G mutation, i.e. d(p*GGCGAGCTAT) (10mer-A) and d(p*GGCGGGCTAT) (10mer-G). However, the corresponding product harboring a T→C mutation at the lesion site, i.e. d(p*GGCGCGCTAT) (10mer-C), could not be separated from the non-mutagenic 10mer-T (Figure 1C and Supplementary Figures S33–S39). On the other hand, the respective radiolabeled complementary strand, i.e. d(p*AATTATAGCN), with ‘N’ being ‘A’ or ‘G’, obtained from sequential digestion with MluCI and BbsI, could be readily resolved from each other (Figure 1B and Supplementary Figures S33–S39). Thus, with the combination of the two enzyme digestion procedures, we were able to distinguish unequivocally the four potential types of replication products, and by monitoring the products from the strand of d(p*AATTATAGCN), we could quantify the frequencies of T→C mutation. It is worth noting that our assay is also capable of detecting putative −1 or −2 frame-shift mutation products; such putative frameshift mutations emanating from the replication of any of the *O*^4^-alkyldT-bearing genomes were, however, below the detection limit of our method. In this vein, it is of note that large deletions resulting in the loss of MluCI and/or BbsI recognition site will not give rise to the restriction fragments used for this assay, and are thus treated as lack of replicative bypass.

The identities of the above restriction digestion products were also confirmed by LC-MS/MS analyses. In particular, we monitored the higher-resolution ‘ultra-zoom scan’ MS for the [M-3H]^3−^ ions of d(GGCGMGCTAT) and d(AATTATAGCN) and their MS/MS (Supplementary Figures S29–S32), where ‘M’ and ‘N’ designate the nucleotides inserted at the initial damage site and the opposing nucleotide in the complementary strand, respectively. Unlike the promiscuous nucleotide misinsertions directed by the minor-groove *O*^2^-alkyldT lesions (39), our results revealed that the eight *O*^4^-alkyldT lesions exclusively induced T→C transition mutation, which is consistent with the previously published results (23). Along this line, our previous study showed that the sensitivity of our LC-MS- and MS/MS-based assay can allow for detection of a mutation frequency that is as low as 0.2% (33).

**Figure 2.**
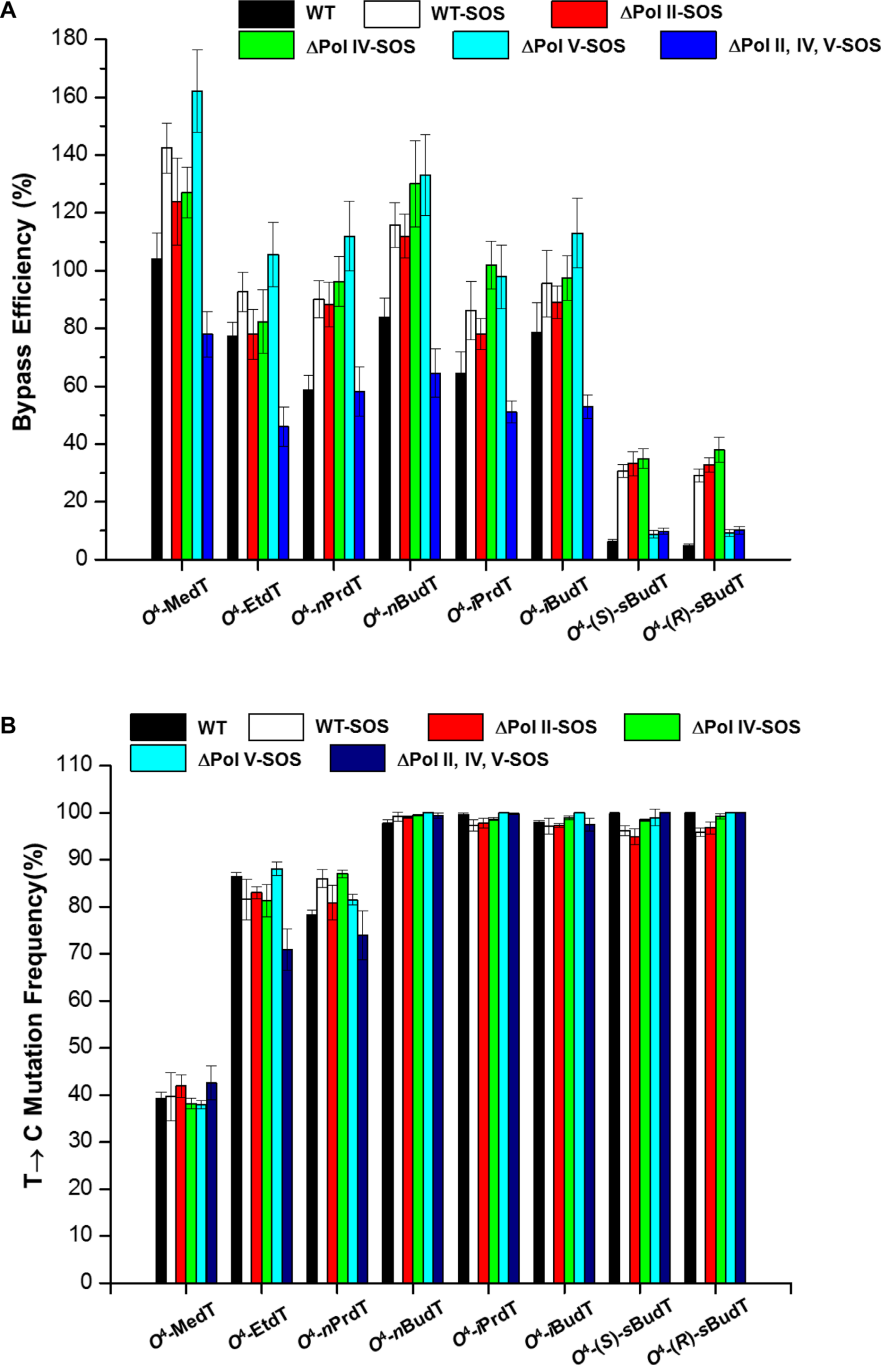
The bypass efficiencies (**A**) and mutation frequencies (**B**) of the *O*^4^-alkyldT lesions in AB1157 *Escherichia coli* strains that are proficient in translesion synthesis or deficient in Pol II, Pol IV, Pol V or all three SOS-induced DNA polymerases. The data represent the means and standard deviations of results from three independent replication experiments.

The bypass efficiencies of the *O*^4^-alkyldT lesions were then calculated from the ratio of the combined intensities of bands observed for the 10-mer products from the lesion-containing genome over the intensity of the 13-mer product from the competitor genome, with the consideration of the molar ratio of the lesion over competitor genomes employed in the initial transfection. The bypass efficiencies for the lesion-carrying genomes were then normalized against that for the control lesion-free genome as described in the ‘Materials and Methods’ section. It turned out that, except for the two diastereomers of *O*^4^-*s*BudT, whose replication bypass efficiencies are ∼5%, other *O*^4^-alkyldT lesions are not strong impediments to DNA replication in wild-type AB1157 cells (Figure 2A). In addition, the bypass efficiencies for the two diastereomers of *O*^4^-*s*BudT are very similar in wild-type AB1157 cells (Figure 2A).

We also examined the roles of the SOS-induced DNA polymerases in bypassing the *O*^4^-alkyldT lesions by conducting the replication experiments in *E. coli* strains deficient in these DNA polymerases. Our results showed that, similar to what we found previously for *O*^4^-EtdT (20), no significant differences in bypass efficiency were observed for all *O*^4^-alkyldT lesions when any of the three SOS-induced polymerases were individually depleted. However, a marked reduction in bypass efficiency was observed for the triple knockout cells (Figure 2A), indicating that Pol II, Pol IV and Pol V play somewhat redundant roles in bypassing the *O*^4^-alkyldT lesions in *E. coli* cells.

The results from native PAGE analysis also allowed us to measure the mutation frequencies of *O*^4^-alkyldT lesions in wild-type and DNA polymerase-deficient AB1157 *E. coli* strains (Figure 2B). Our results revealed that all *O*^4^-alkyldT lesions are highly mutagenic, resulting exclusively in T→C mutation at frequencies of up to nearly 100%. Interestingly, the mere increase in the size of the alkyl group from methyl to ethyl led to a two-fold elevation in the T→C mutation frequency (i.e. from ∼40 to 80%). It is also worth noting that single or even triple depletion of these three polymerases did not confer any significant change in mutation frequencies, suggesting that the miscoding property of the *O*^4^-alkyldT lesions is independent of the nature of polymerases involved.

**Figure 3.**
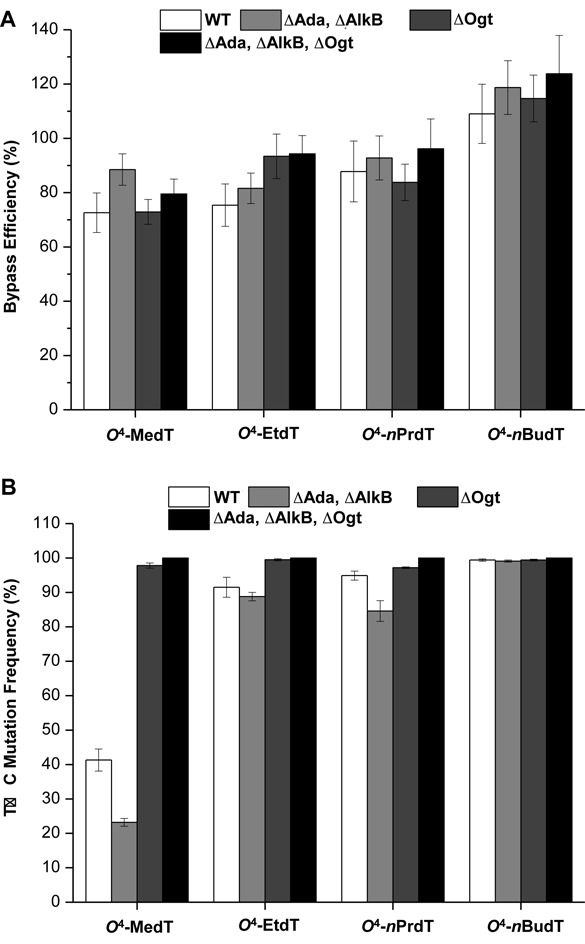
The bypass efficiencies (**A**) and and mutation frequencies (**B**) of the *O*^4^-alkyldT lesions in SOS-induced *Escherichia coli* cells that are proficient in *O*^6^-alkylguanine-DNA alkyltransferases or deficient in Ogt (ΔOgt), both Ada and Ogt (ΔAda, ΔOgt), or Ada, AlkB and Ogt (ΔAda, ΔAlkB, ΔOgt). The data represent the means and standard deviations of results from three independent replication experiments.

The above replication experiments showed that mutation frequencies for the *O*^4^-alkyldT lesions with small straight-chain alkyl groups (i.e., *O*^4^-MedT, *O*^4^-EtdT and *O*^4^-*n*PrdT) were not as high as the other lesions investigated. This might be attributed to the differential repair of the *O*^4^-alkyldT lesions. To test this, we exploited the involvement of *O*^6^-alkylguanine-DNA alkyltransferases in the repair of *O*^4^-MedT, *O*^4^-EtdT, *O*^4^-*n*PrdT and *O*^4^-*n*BudT lesions by conducting the replication experiments in isogenic *E. coli* cells that are proficient or deficient in Ogt and/or Ada (Figure 3). We found that the bypass efficiencies for the four *O*^4^-alkyldT lesions were not significantly altered upon the depletion of these DNA repair factors (Figure 3A). However, the removal of Ogt in the wild-type or Ada/AlkB-double knockout background gave rise to elevations in T→C mutation frequencies of *O*^4^-MedT, *O*^4^-EtdT and *O*^4^-*n*PrdT to nearly 100%, supporting the involvement of Ogt in the repair of these lesions (Figure 3B). Dual depletion of Ada and AlkB in wild-type or Ogt-deficient background, however, did not result in any appreciable increase in mutation frequencies for any of the *O*^4^-alkyldT lesions, suggesting the lack of involvement of Ada in their repair. The lower mutation frequency of *O*^4^-MedT than *O*^4^-EtdT and *O*^4^-*n*PrdT in wild-type cells also reveals that the Ogt-mediated repair of *O*^4^-MedT is more efficient than *O*^4^-EtdT and *O*^4^-*n*PrdT.

## DISCUSSION

We systematically investigated the cytotoxic and mutagenic properties of the *O*^4^-alkyldT lesions in *E. coli* cells and our results led to several important findings. First, we observed that all *O*^4^-alkyldT lesions except for (*S*)- and (*R*)-*O*^4^-*s*BudT do not strongly inhibit DNA replication in *E. coli* cells. However, the two diastereomers of *O*^4^-*s*BudT are still bypassed more efficiently than simple *O*^2^-MedT (39), suggesting that the major-groove pyrimidine lesions are much better tolerated than the minor-groove counterparts by the *E. coli* DNA replication machinery.

Second, our results demonstrated that the nucleotide insertion opposite *O*^4^-alkyldT lesions is specific, where only T→C mutation was observed for all the eight *O*^4^-alkyldT lesions and the mutation frequencies of the *O*^4^-alkyldT lesions were not affected by depletion of Pol II, Pol IV, Pol V or all three polymerases. The mutation frequencies were also not perturbed by SOS induction in wild-type AB 1157 cells, indicating that the selective dGMP misinsertion may be attributed to the distinct chemical properties of the *O*^4^-alkyldT lesions. In this vein, the addition of a methyl group to *O*^4^ position of thymine is known to facilitate its favorable base pairing with guanine (9,40).

Third, our results revealed that the three SOS-induced DNA polymerases play somewhat redundant roles in bypassing the *O*^4^-alkyldT lesions with the exception of (*S*)- and (*R*)-*O*^4^-*s*BudT, as reflected by the observation that the bypass efficiencies of these lesions dropped substantially only upon depletion of all three polymerases. Efficient bypass of *O*^4^-*s*BudT lesions, however, necessitates Pol V. In this connection, Yuan *et* *al*. (41) observed that Pol V also plays an important role in the bypass of *O*^4^-carboxymethyl-dT in *E. coli* cells.

Fourth, we found that Ogt, but not Ada, is involved in repairing *O*^4^-alkyldT with small straight-chain alkyl functionality, with *O*^4^-MedT being much more efficiently repaired than *O*^4^-EtdT and *O*^4^-*n*PrdT. The nearly 100% T→C mutation frequency observed for *O*^4^-*i*PrdT, *O*^4^-*n*BudT, *O*^4^-*i*BudT or *O*^4^-*s*BudT suggest the lack of repair of these lesions in *E. coli* cells. This observation is in line with the previous observations that Ada is not involved in repairing *O*^4^-alkyldT and that Ogt is slightly better than Ada in tolerating large *O*^6^-alkyldG adducts (17,29,30). Likewise, all the eight *O*^4^-alkyldT lesions were completely mutagenic in Ogt-deficient *E. coli* cells, suggesting the absence of other DNA repair mechanisms in the removal of these DNA lesions when they are situated in single-stranded M13 genome. In this context, it is important to note that the replication of these lesions in the single-stranded M13 genome differs from the replication of these lesions in double-stranded chromosomal DNA in *E. coli* cells. In particular, some DNA repair machinery (e.g. nucleotide excision repair) necessitates double-stranded DNA substrates; thus, the modulation of mutation frequencies of these lesions by some DNA repair activities may not be revealed from the replication experiments with the use of single-stranded plasmid.

Different from the minor-groove *O*^2^-alkyldT lesions (39), the bypass efficiencies of the major-groove *O*^4^-alkyldT do not exhibit a clear trend with the sizes of alkyl groups attached to the *O*^4^ position of thymine, which could be attributed to the lack of perturbation of base pairing between *O*^4^-alkyldT and dG by the substituent groups on the *O*^4^ of thymine (9,40). The failure to observe a marked decrease in bypass efficiency with the increase of the size of the alkyl group also suggests that the active sites of polymerases are spacious enough to accommodate the majority of the alkyl groups situated on the *O*^4^ position of thymine. However, the two diastereomers of *O*^4^-*s*BudT significantly blocked DNA replication, suggesting that a branched ethyl group, but not a methyl group (as in *O*^4^-*i*PrdT), located at the α-carbon of the *O*^4^ of thymine may render it difficult for the modified thymine to fit into the active sites of DNA polymerases.

In summary, our systematic shuttle vector-based study of a group of structurally defined *O*^4^-alkyldT lesions provided important new insights into the impact of this group of DNA lesions on the efficiency and accuracy of DNA replication and the repair of these lesions *in vivo*. Our results also indicated that the carcinogenic potentials of these lesions may arise, at least in part, from their high bypass efficiencies and strong mutagenic effects. It will be important to assess how the *O*^4^-alkyldT lesions compromise DNA replication and how they are repaired in mammalian cells in the future.

## Supplementary Material

SUPPLEMENTARY DATA
