# Supplementary material for: Cytotoxic and mutagenic properties of O4-alkylthymidine lesions in Escherichia coli cells
Source: Nucleic Acids Res. 2015 Sep 22;43(22):10795–803. doi: 10.1093/nar/gkv941 (PMC4678858; doi:10.1093/nar/gkv941)
Supplement: SUPPLEMENTARY DATA [file supp_43_22_10795__index.html]

Cytotoxic and mutagenic properties of O4-alkylthymidine lesions in Escherichia coli cells — Cytotoxic and mutagenic properties of O4-alkylthymidine lesions in Escherichia coli cells — SUPPLEMENTARY DATA 

# Cytotoxic and mutagenic properties of *O*4-alkylthymidine lesions in *Escherichia coli* cells

## SUPPLEMENTARY DATA

- SUPPLEMENTARY DATA
