# Supplementary material for: Cytotoxic and mutagenic properties of O4-alkylthymidine lesions in Escherichia coli cells
Source: Nucleic Acids Res. 2015 Sep 22;43(22):10795–803. doi: 10.1093/nar/gkv941 (PMC4678858; doi:10.1093/nar/gkv941)
Supplement: SUPPLEMENTARY DATA [file supp_gkv941_nar-01775-d-2015-File002.pdf]

Supplementary Materials for

"Cytotoxic and Mutagenic Properties of *O*^{4}-Alkylthymidine Lesions in *Escherichia coli* cells"

by Wang, P. et al. *Nucleic Acids Res.*, 2015

Supplementary Materials and Methods:

Mass Spectrometry (MS) and NMR

Electrospray ionization-MS (ESI-MS) and tandem MS (MS/MS) experiments were carried out on an LCQ Deca XP ion-trap mass spectrometer (Thermo Fisher Scientific, San Jose, CA). A mixture of acetonitrile and water (50:50, v/v) was used as the solvent for electrospray. The spray voltage was 3.0 kV, and the temperature of the ion transport tube was maintained at 275°C. High-resolution mass spectra (HRMS) were acquired on an Agilent 6510 Q-TOF LC/MS instrument (Agilent Technologies, Palo Alto, CA) equipped with an electrospray ionization (ESI) source. ^{1}H NMR spectra were recorded at 300 MHz on a Varian Inova 300 NMR spectrometer (Varian Inc., Palo Alto, CA), and ^{31}P NMR spectra were acquired at 80 MHz on the same instrument.

Reaction yields, and NMR and mass spectrometric characterizations of the synthetic products:

***O*^{4}-*n*-propylthymidine (*O*^{4}-*n*PrdT, 2c)**. Obtained as a white solid (67% yield); ^{1}H NMR (300 MHz, CD_3OD) δ 8.13 (s, 1H), 6.24 (t, *J* = 6.4 Hz, 1H), 4.39 (dt, *J* = 6.3, 3.9 Hz, 1H), 4.32 (t, *J* = 6.6 Hz, 2H), 3.96 (dd, *J* = 7.0, 3.6 Hz, 1H), 3.80 (ddd, *J* = 28.2, 12.1, 3.4 Hz, 2H), 2.41 (ddd, *J* = 13.5, 6.2, 4.1 Hz, 1H), 2.21 – 2.09 (m, 1H), 1.98 (s, 3H), 1.86 – 1.72 (m, 2H), 1.02 (t, J = 7.4 Hz, 3H). HRMS (ESI) calcd for $C_{13}H_{21}N_2O_5$ $[M+H]^+$ 285.1445, found 285.1446.

***O*^{4}-*iso*-propylthymidine (*O*^{4}-*i*PrdT, 2d)**. Obtained as white solid (49% yield); ^{1}H NMR (300 MHz, CD_3OD) δ 8.11 (s, 1H), 6.25 (t, *J* = 6.4 Hz, 1H), 5.38 (dd, *J* = 12.4, 6.2 Hz, 1H), 4.42 – 4.34 (m, 1H), 3.96 (dd, *J* = 6.9, 3.5 Hz, 1H), 3.79 (ddd, *J* = 28.3, 12.1, 3.4 Hz, 2H), 2.41 (ddd, *J* = 13.5, 6.1, 4.1 Hz, 1H), 2.20 – 2.11 (m, 1H), 1.95 (s, 3H), 1.36 (s, 3H), 1.34 (s, 3H). HRMS (ESI) calcd for $C_{13}H_{21}N_2O_5$ $[M+H]^+$ 285.1445, found 285.1458.

***O*^{4}-*n*-butylthymidine (*O*^{4}-*n*BudT, 2e)**. Obtained as colorless film (60% yield); ^{1}H NMR (300 MHz, CD_3OD) δ 8.13 (s, 1H), 6.24 (t, *J* = 6.4 Hz, 1H), 4.42 – 4.32 (m, 3H), 3.96 (q, *J* = 3.5 Hz, 1H), 3.80 (ddd, *J* = 28.1, 12.1, 3.4 Hz, 2H), 2.41 (ddd, *J* = 13.6, 6.1, 4.1 Hz, 1H), 2.19 – 2.11 (m, 1H), 1.97 (s, 3H), 1.81 – 1.70 (m, 2H), 1.54 – 1.40 (m, 2H), 0.98 (t, *J* = 7.4 Hz, 3H). HRMS (ESI) calcd for $C_{14}H_{23}N_2O_5$ $[M+H]^+$ 299.1601, found 299.1604.

***O*^{4}-*iso*-butylthymidine (*O*^{4}-*i*BudT, 2f)**. Obtained as colorless film (58% yield); ^{1}H NMR (300 MHz, CD_3OD) δ 8.13 (s, 1H), 6.24 (t, *J* = 6.3 Hz, 1H), 4.41 – 4.35 (m, 1H), 4.14 (d, *J* = 6.5 Hz, 2H), 3.96 (q, *J* = 3.3 Hz, 1H), 3.80 (ddd, *J* = 28.5, 12.1, 3.3 Hz, 2H), 2.41 (ddd, *J* = 13.5, 6.1, 4.1 Hz, 1H), 2.19 – 2.04 (m, 2H), 1.99 (s, 3H), 1.03 (s, 3H), 1.00 (s, 3H). HRMS (ESI) calcd for $C_{14}H_{23}N_2O_5$ $[M+H]^+$ 299.1601, found 299.1613.

***O*^{4}-(*S*)-*sec*-butylthymidine (*O*^{4}-(*S*)-*s*BudT, 2g)**. Obtained as colorless film (53% yield); ^{1}H NMR (300 MHz, CD_3OD) δ 8.12 (s, 1H), 6.25 (t, *J* = 6.3 Hz, 1H), 5.26 (dd, *J* = 12.2, 6.0 Hz, 1H), 4.41 – 4.35 (m, 1H), 3.98 – 3.94 (m, 1H), 3.80 (ddd, *J* = 15.2, 12.0, 2.7 Hz, 2H), 2.47 – 2.34 (m, 1H), 2.22 – 2.09 (m, 1H), 1.96 (s, 3H), 1.80 – 1.59 (m, 2H), 1.32 (d, *J* = 6.2 Hz, 3H), 0.96 (t, *J* = 7.4 Hz, 3H). HRMS (ESI) calcd for $C_{14}H_{23}N_2O_5$ $[M+H]^+$ 299.1601, found 299.1606.

O^4-(*R*)-*sec*-butylthymidine (O^4-(*R*)-*s*BudT, 2h). Obtained as colorless film (52% yield); ^{1}H NMR (300 MHz, CD_3OD) δ 8.12 (s, 1H), 6.25 (t, *J* = 6.4 Hz, 1H), 5.25 (dd, *J* = 12.3, 6.1 Hz, 1H), 4.39 (dt, *J* = 6.4, 3.9 Hz, 1H), 3.96 (q, *J* = 3.6 Hz, 1H), 3.80 (ddd, *J* = 28.2, 12.1, 3.4 Hz, 2H), 2.41 (ddd, *J* = 13.5, 6.1, 4.1 Hz, 1H), 2.21 – 2.10 (m, 1H), 1.96 (s, 3H), 1.80 – 1.62 (m, 2H), 1.32 (d, *J* = 6.2 Hz, 3H), 0.95 (t, *J* = 7.4 Hz, 3H). HRMS (ESI) calcd for $C_{14}H_{23}N_2O_5$ $[M+H]^+$ 299.1601, found 299.1621.

5'-*O*-(4,4'-dimethoxyltrityl)-O^4-*n*-propylthymidine (DMTr-O^4-*n*PrdT, 3c). Obtained as white foam (62% yield). ^{1}H NMR (300 MHz, acetone-d_6) δ 7.90 (s, 1H), 7.52 – 7.46 (m, 2H), 7.41 – 7.23 (m, 7H), 6.91 (dd, *J* = 8.8, 1.4 Hz, 4H), 6.32 (t, *J* = 6.5 Hz, 1H), 4.57 (s, 1H), 4.26 (td, *J* = 6.6, 1.5 Hz, 2H), 4.12 – 4.02 (m, 1H), 3.80 (s, 6H), 3.40 (d, *J* = 2.8 Hz, 2H), 2.50 – 2.39 (m, 1H), 2.32 – 2.21 (m, 1H), 1.82 – 1.70 (m, 2H), 1.59 (s, 3H), 0.99 (td, *J* = 7.4, 1.5 Hz, 3H). HRMS (ESI) calcd for $C_{34}H_{38}N_2O_7Na$ $[M+Na]^+$ 609.2571, found 609.2585.

5'-*O*-(4,4'-dimethoxyltrityl)-O^4-*iso*-propylthymidine (DMTr-O^4-*i*PrdT, 3d). Obtained as white foam (54% yield). ^{1}H NMR (300 MHz, acetone-d_6) δ 7.89 (s, 1H), 7.53 – 7.47 (m, 2H), 7.40 – 7.23 (m, 7H), 6.91 (dd, *J* = 8.8, 1.4 Hz, 4H), 6.33 (t, *J* = 6.5 Hz, 1H), 5.35 (dt, *J* = 12.4, 6.2 Hz, 1H), 4.61 – 4.55 (m, 1H), 4.12 – 4.05 (m, 1H), 3.80 (s, 6H), 3.41 (d, *J* = 3.3 Hz, 2H), 2.44 (ddd, *J* = 13.5, 6.1, 3.8 Hz, 1H), 2.26 (dt, *J* = 13.3, 6.6 Hz, 1H), 1.56 (s, 3H), 1.33 (d, *J* = 2.0 Hz, 3H), 1.31 (d, *J* = 1.9 Hz, 3H). HRMS (ESI) calcd for $C_{34}H_{38}N_2O_7Na$ $[M+Na]^+$ 609.2571, found 609.2596.

5'-*O*-(4,4'-dimethoxyltrityl)-O^4-*n*-butylthymidine (DMTr-O^4-*n*BudT, 3e). Obtained as white foam (64% yield). ^{1}H NMR (300 MHz, CD_3OD) δ 8.01 (s, 1H), 7.46 – 7.40 (m, 2H), 7.34 – 7.21 (m, 7H), 6.86 (d, *J* = 8.9 Hz, 4H), 6.27 (t, *J* = 6.2 Hz, 1H), 4.51 (dt, *J* = 6.1, 4.0 Hz, 1H), 4.36 (t, *J* = 6.5 Hz, 2H), 4.11 – 4.07 (m, 1H), 3.78 (s, 6H), 3.48 – 3.34 (m, 2H), 2.56 – 2.44 (m, 1H), 2.37 – 2.25 (m, 1H), 1.82 – 1.70 (m, 2H), 1.51 (s, 3H), 1.49 – 1.40 (m, 2H), 0.98 (t, J = 7.4 Hz, 3H). HRMS (ESI) calcd for $C_{35}H_{40}N_2O_7Na$ $[M+H]^+$ 623.2728, found 623.2758.

5'-*O*-(4,4'-dimethoxyltrityl)-O^4-*iso*-butylthymidine (DMTr-O^4-*i*BudT, 3f). Obtained as white foam (65% yield). ^{1}H NMR (300 MHz, acetone-d_6) δ 7.91 (s, 1H), 7.52 – 7.47 (m, 2H), 7.40 – 7.23 (m, 7H), 6.91 (d, *J* = 8.9 Hz, 4H), 6.32 (t, *J* = 6.5 Hz, 1H), 4.58 (dt, *J* = 6.7, 3.8 Hz, 1H), 4.12 – 4.07 (m, 3H), 3.80 (s, 6H), 3.41 (d, *J* = 3.5 Hz, 2H), 2.45 (ddd, *J* = 13.4, 6.1, 3.9 Hz, 1H), 2.27 (dt, *J* = 13.4, 6.6 Hz, 1H), 2.13 – 2.08 (m, 1H), 1.61 (s, 3H), 1.01 (s, 3H), 0.99 (s, 3H). HRMS (ESI) calcd for $C_{35}H_{40}N_2O_7Na$ $[M+Na]^+$ 623.2728, found 623.2736.

5'-*O*-(4,4'-dimethoxyltrityl)-O^4-(*S*)-*sec*-butylthymidine (DMTr-O^4-(*S*)-*s*BudT, 3g). Obtained as white foam (63% yield). ^{1}H NMR (300 MHz, acetone-d_6) δ 7.89 (s, 1H), 7.53 – 7.45 (m, 2H), 7.42 – 7.20 (m, 7H), 6.91 (d, *J* = 8.8 Hz, 4H), 6.33 (t, *J* = 6.5 Hz, 1H), 5.25 – 5.15 (m, 1H), 4.57 (s, 1H), 4.12 – 4.08 (m, 1H), 3.80 (s, 6H), 3.41 (d, *J* = 3.6 Hz, 2H), 2.45 (ddd, *J* = 13.5, 6.1, 3.8 Hz, 1H), 2.27 (dt, *J* = 13.3, 6.6 Hz, 1H), 1.77 – 1.62 (m, 2H), 1.58 (s, 3H), 1.28 (d, *J* = 6.2 Hz, 3H), 0.94 (t, *J* = 7.4 Hz, 3H). HRMS (ESI) calcd for $C_{35}H_{40}N_2O_7Na$ $[M+Na]^+$ 623.2728, found 623.2749.

5'-*O*-(4,4'-dimethoxyltrityl)-O^4-(*R*)-*sec*-butylthymidine (DMTr-O^4-(*R*)-*s*BudT, 3h). Obtained as white foam (59% yield). ^{1}H NMR (300 MHz, acetone-d_6) δ 7.89 (s, 1H), 7.53 – 7.44 (m, 2H), 7.41 – 7.22 (m, 7H), 6.91 (d, *J* = 8.9 Hz, 4H), 6.33 (t, *J* = 6.5 Hz, 1H), 5.26 –

5.18 (m, 1H), 4.56 (s, 1H), 4.11 – 4.08 (m, 1H), 3.80 (s, 6H), 3.40 (d, *J* = 3.6 Hz, 2H), 2.49 – 2.36 (m, 1H), 2.26 (dt, *J* = 13.2, 6.6 Hz, 1H), 1.77 – 1.63 (m, 2H), 1.58 (s, 3H), 1.29 (d, *J* = 6.2 Hz, 3H), 0.94 (t, *J* = 7.4 Hz, 3H). HRMS (ESI) calcd for $C_{35}H_{40}N_2O_7Na$ $[M+Na]^+$ 623.2728, found 623.2751.

4c: ^{31}P NMR ($CDCl_3$): δ 150.14, 149.52.

4d: ^{31}P NMR ($CDCl_3$): δ 150.10, 149.50.

4e: ^{31}P NMR ($CDCl_3$): δ 150.13, 149.51.

4f: ^{31}P NMR ($CDCl_3$): δ 150.03, 149.49.

4g: ^{31}P NMR ($CDCl_3$): δ 150.08, 149.49.

4h: ^{31}P NMR ($CDCl_3$): δ 150.10, 149.51.

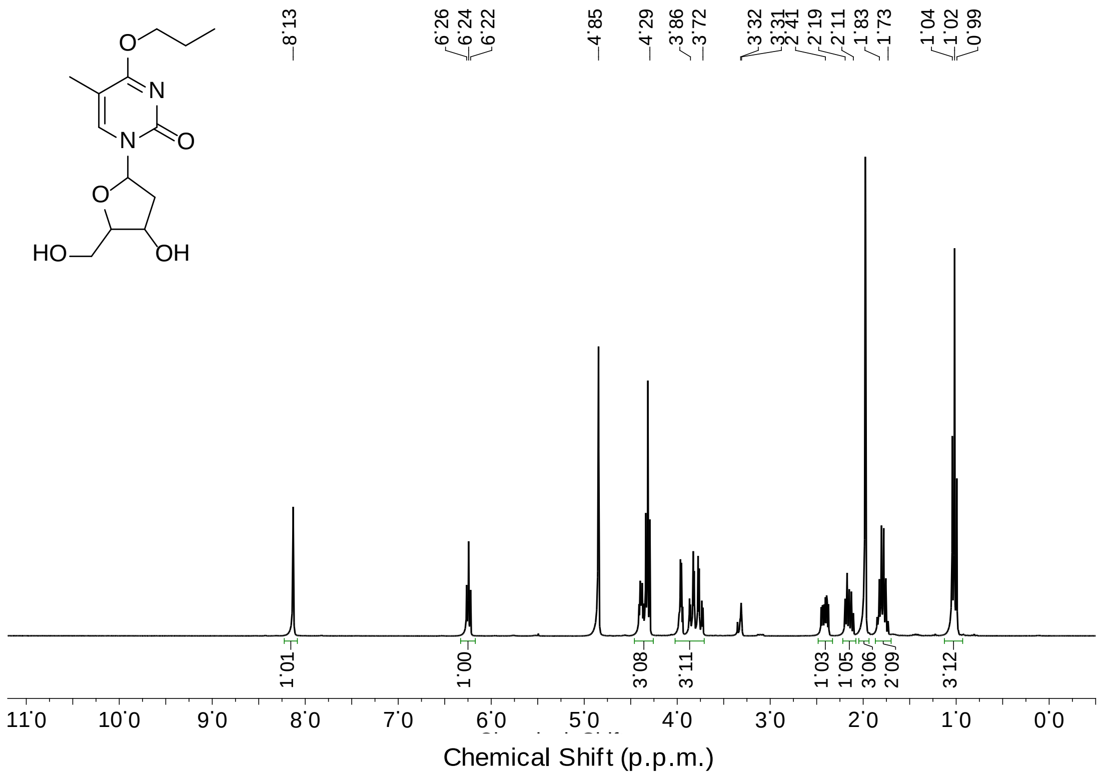

Figure S1. ^{1}H NMR spectrum of O^4-*n*PrdT (**2c**) (300 MHz, CD_3OD, 25°C).

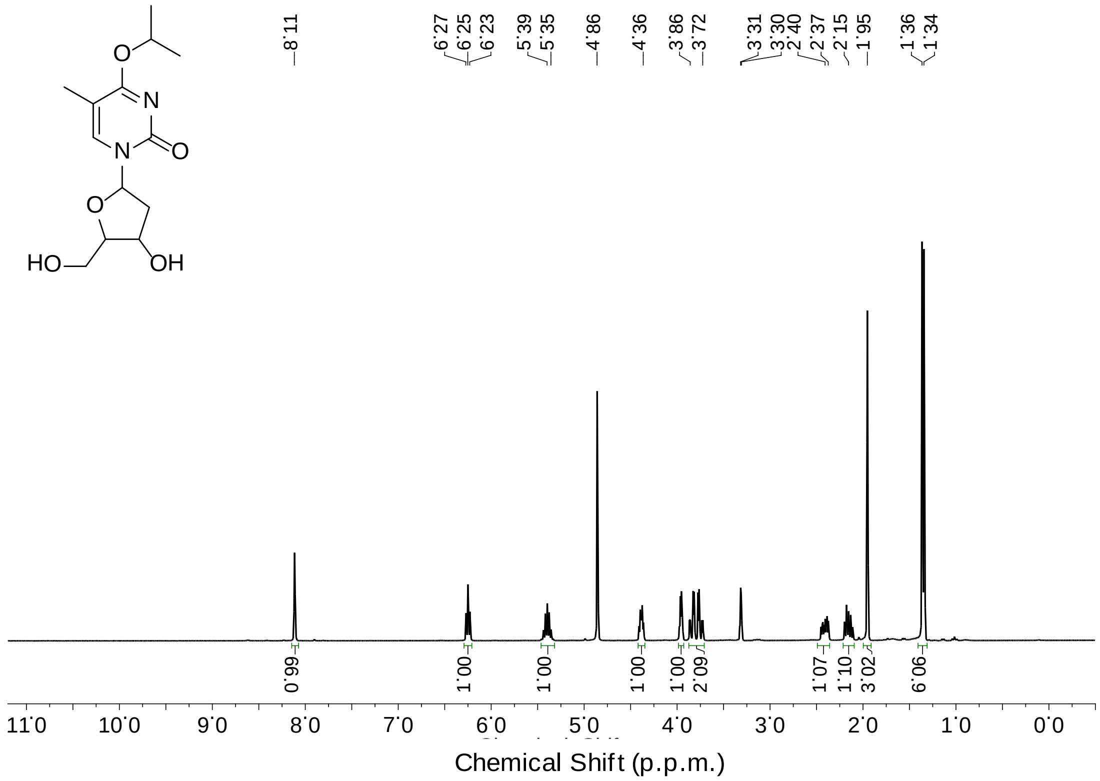

Figure S2. ^{1}H NMR spectrum of O^4-*i*PrdT (**2d**) (300 MHz, CD_3OD, 25°C).

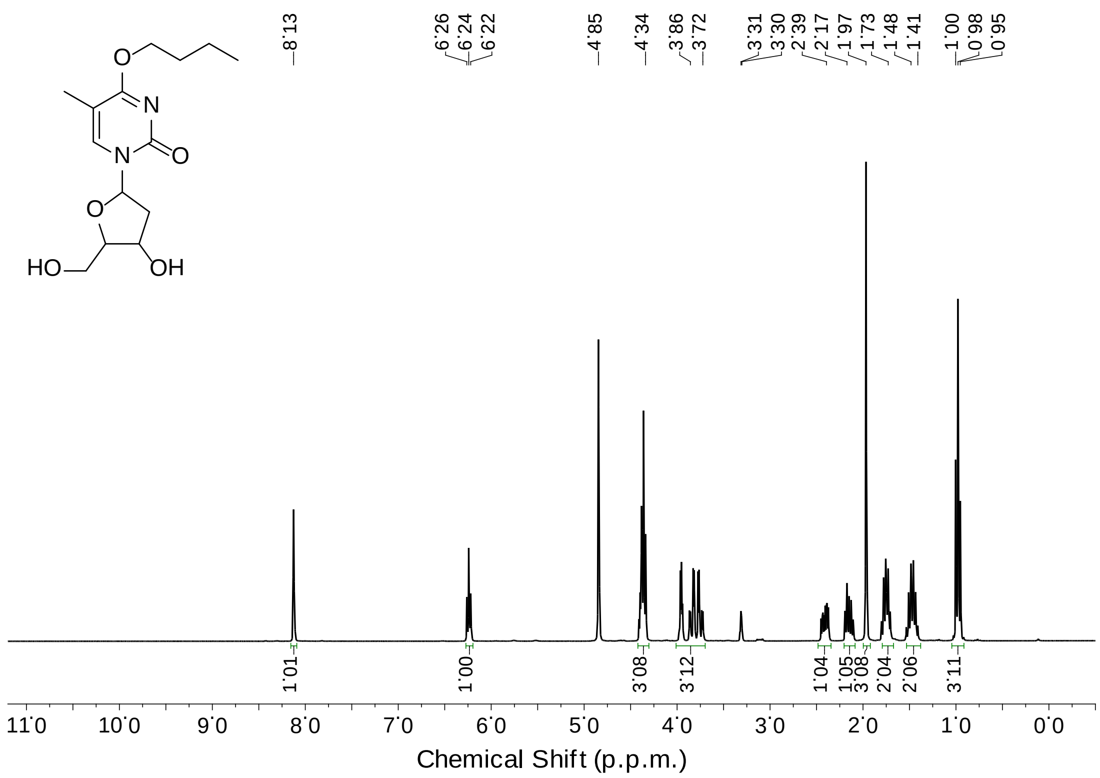

Figure S3. ^{1}H NMR spectrum of *O*[4]-*n*BudT (**2e**) (300 MHz, CD_3OD, 25°C).

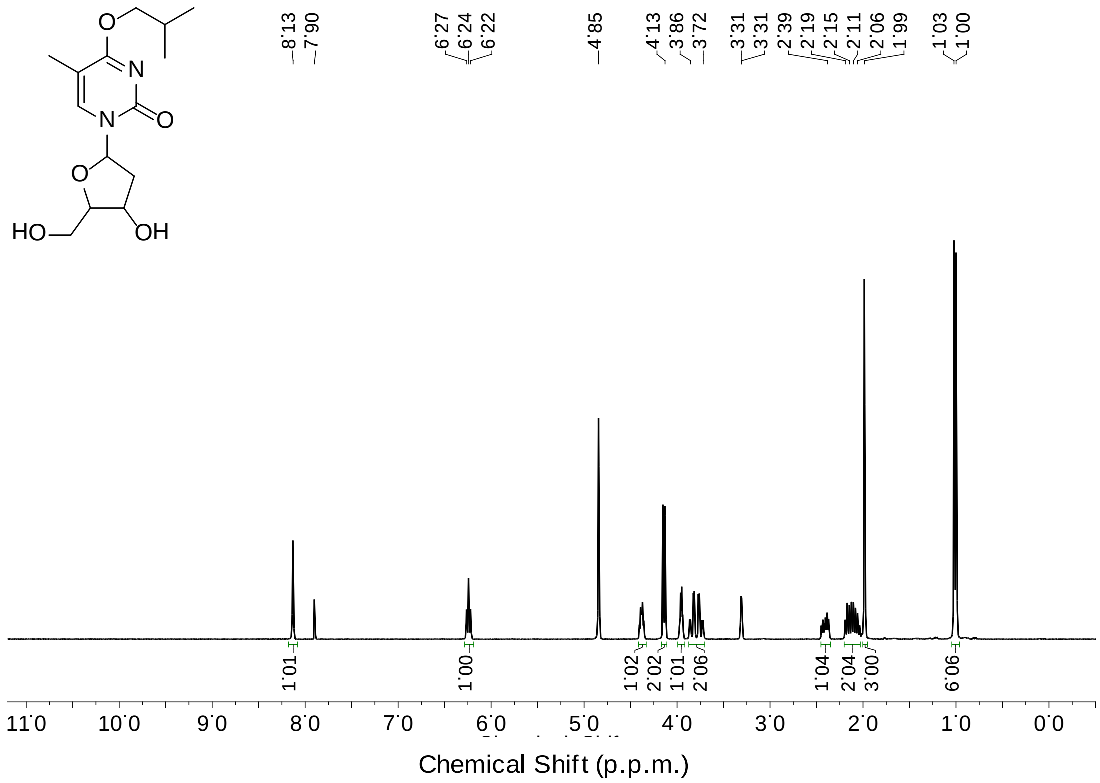

Figure S4. ^{1}H NMR spectrum of *O*[4]-*i*BudT (**2f**) (300 MHz, CD_3OD, 25°C).

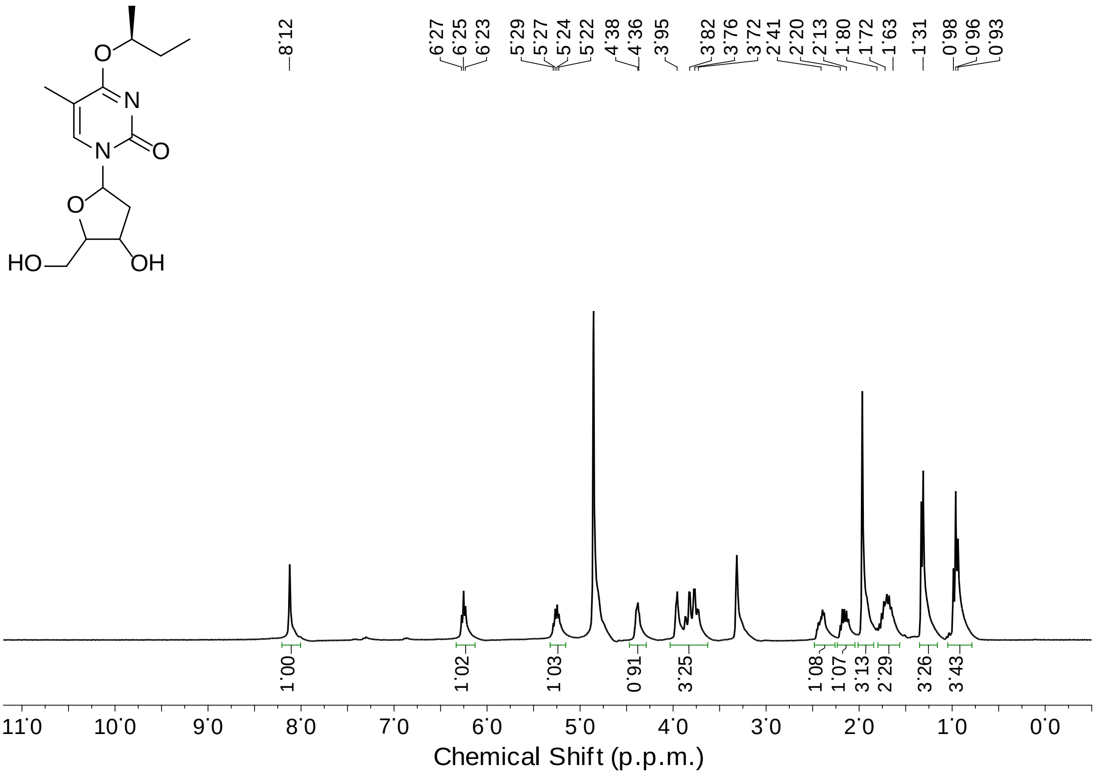

Figure S5. ^{1}H NMR spectrum of *O*^{4}-(*S*)-*s*BudT (**2g**) (300 MHz, CD_3OD, 25°C).

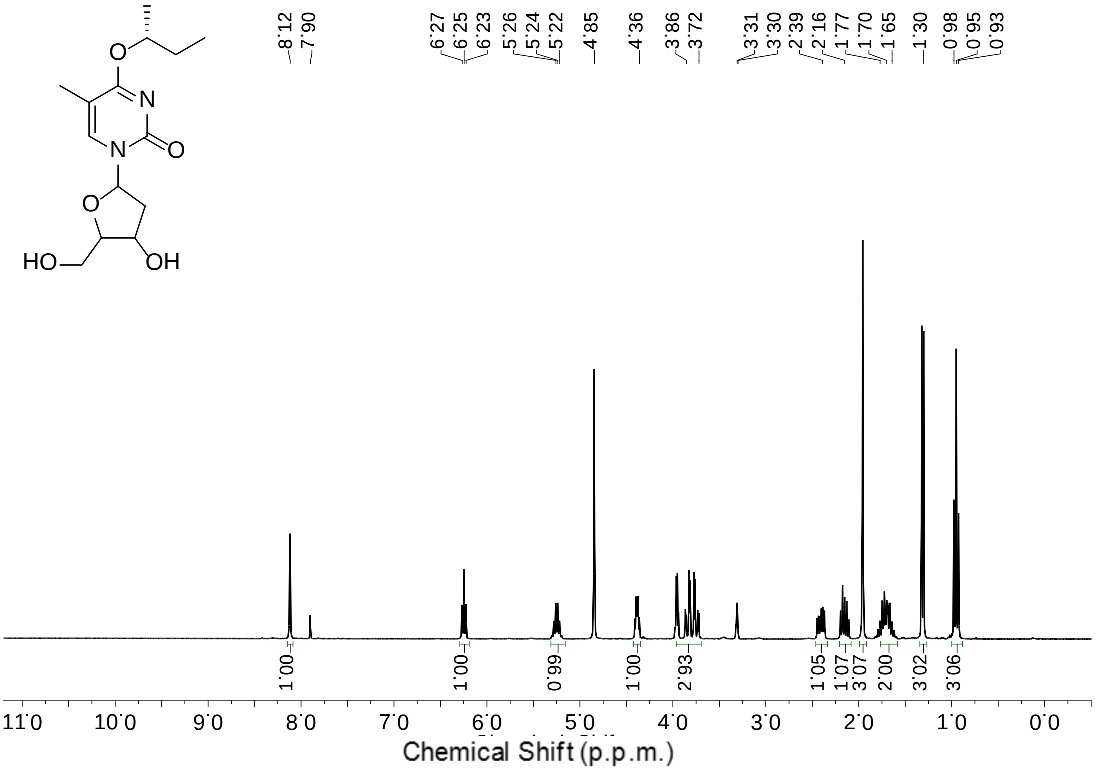

Figure S6. ^{1}H NMR spectrum of *O*^{4}-(*R*)-*s*BudT (**2h**) (300 MHz, CD_3OD, 25°C).

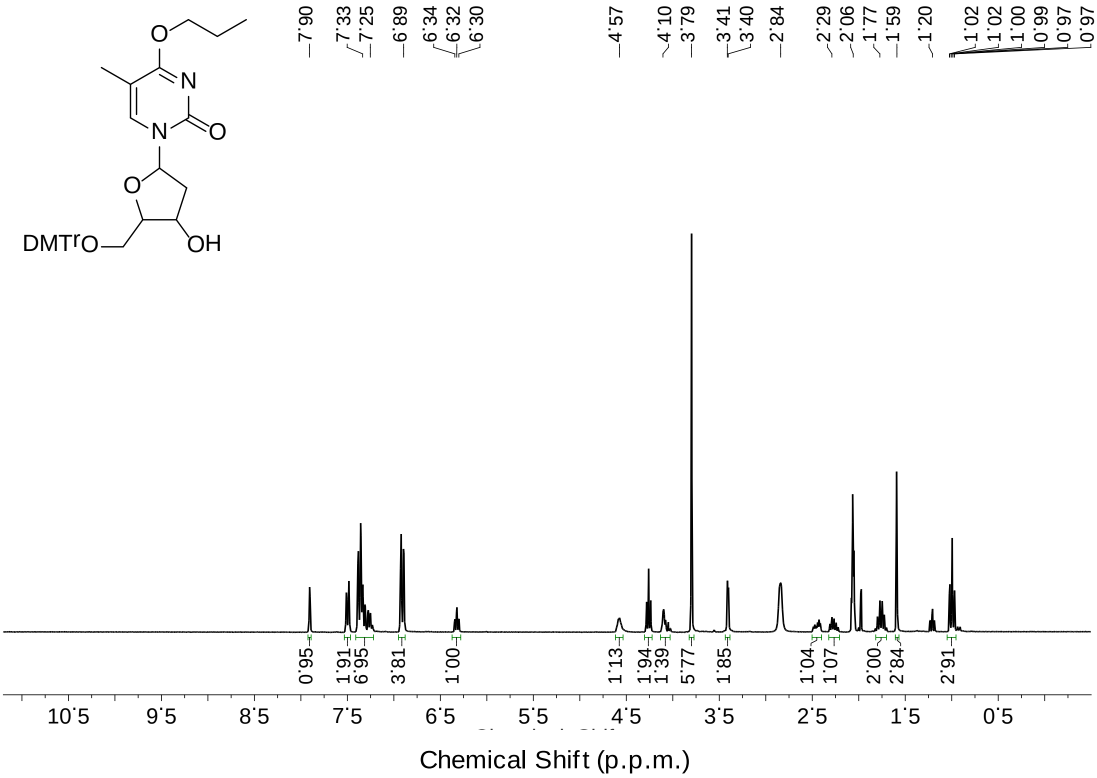

Figure S7. ^{1}H NMR spectrum of *O*^{4}-DMTr-*n*PrdT (**3c**) (300 MHz, acetone-d_6, 25°C).

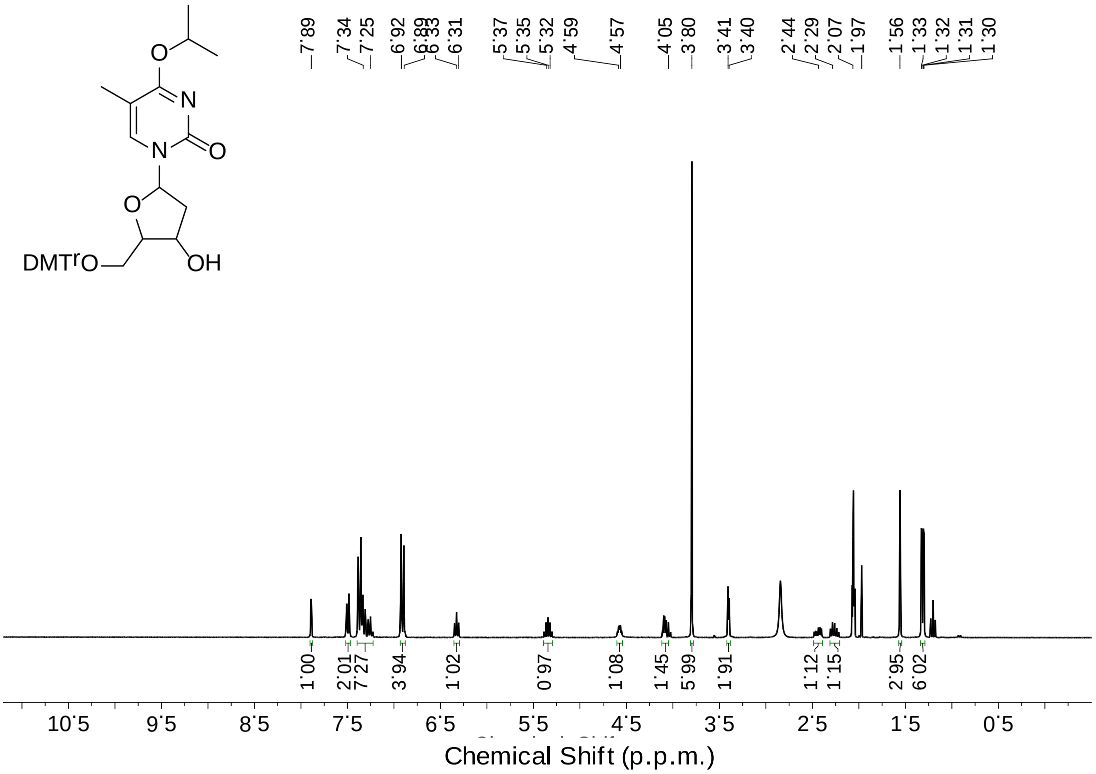

Figure S8. ^{1}H NMR spectrum of *O*^{4}-DMTr-*i*PrdT (**3d**) (300 MHz, acetone-d_6, 25°C).

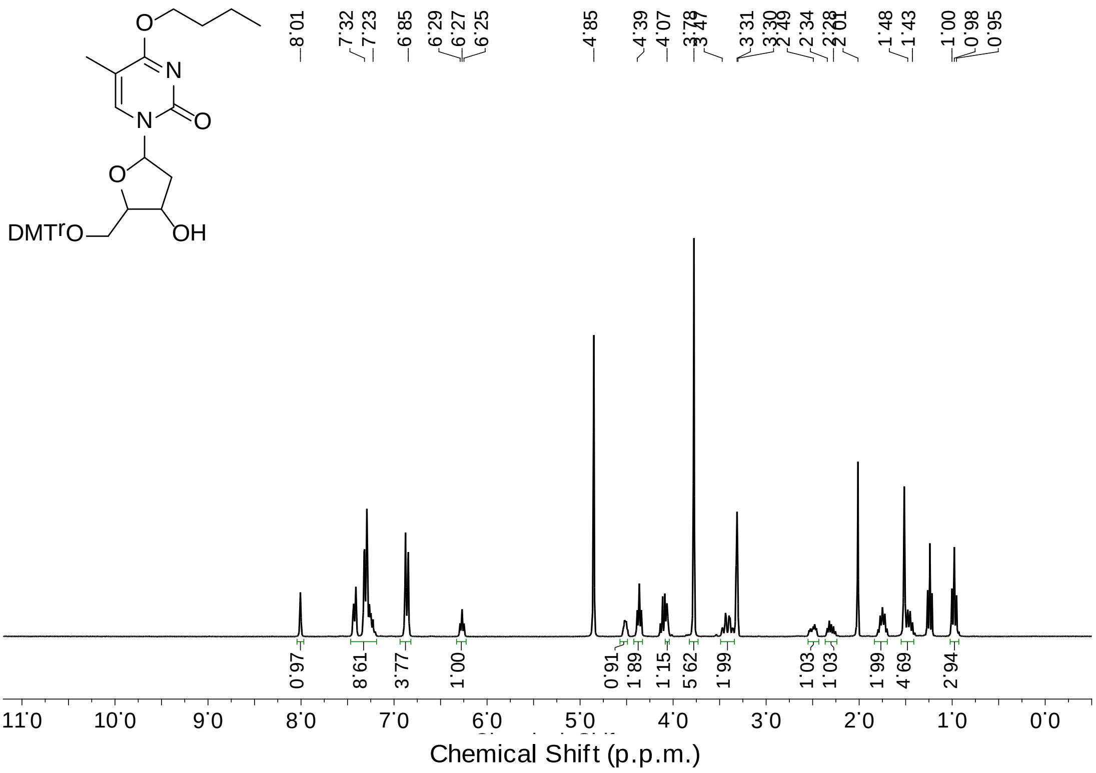

Figure S9. ^{1}H NMR spectrum of *O*[4]-DMTr-*n*BudT (**3e**) (300 MHz, CD_3OD, 25°C).

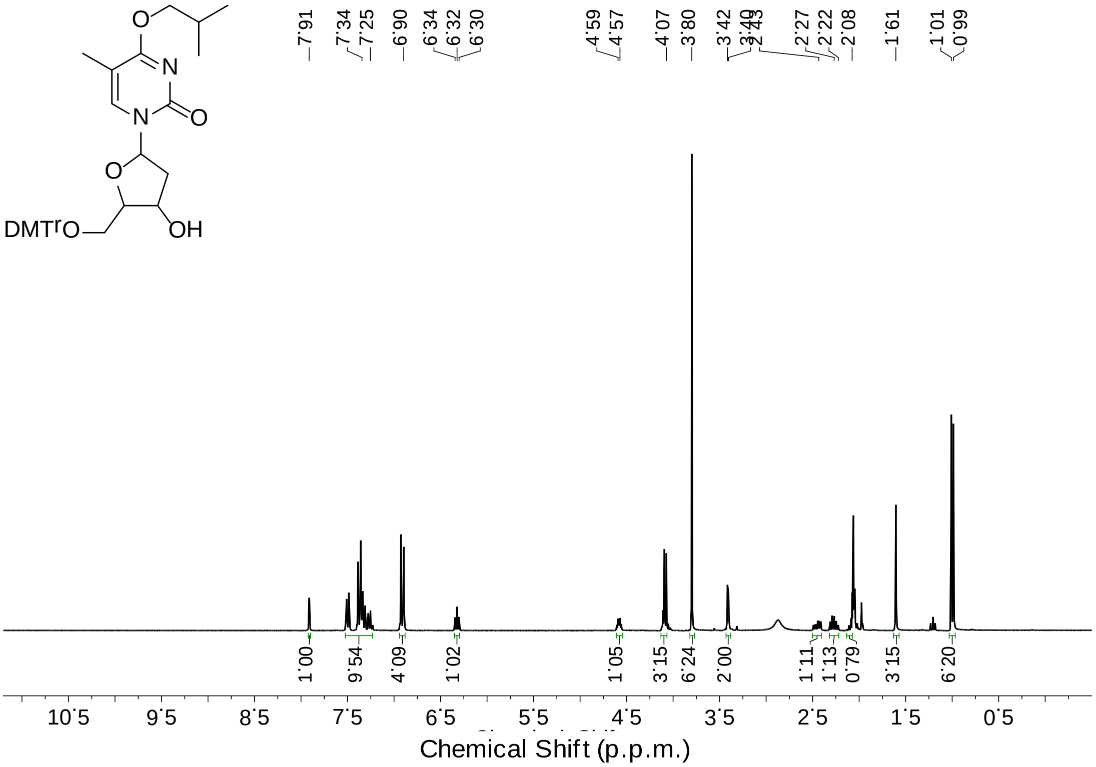

Figure S10. ^{1}H NMR spectrum of *O*[4]-DMTr-*i*BudT (**3f**) (300 MHz, acetone-d_6, 25°C).

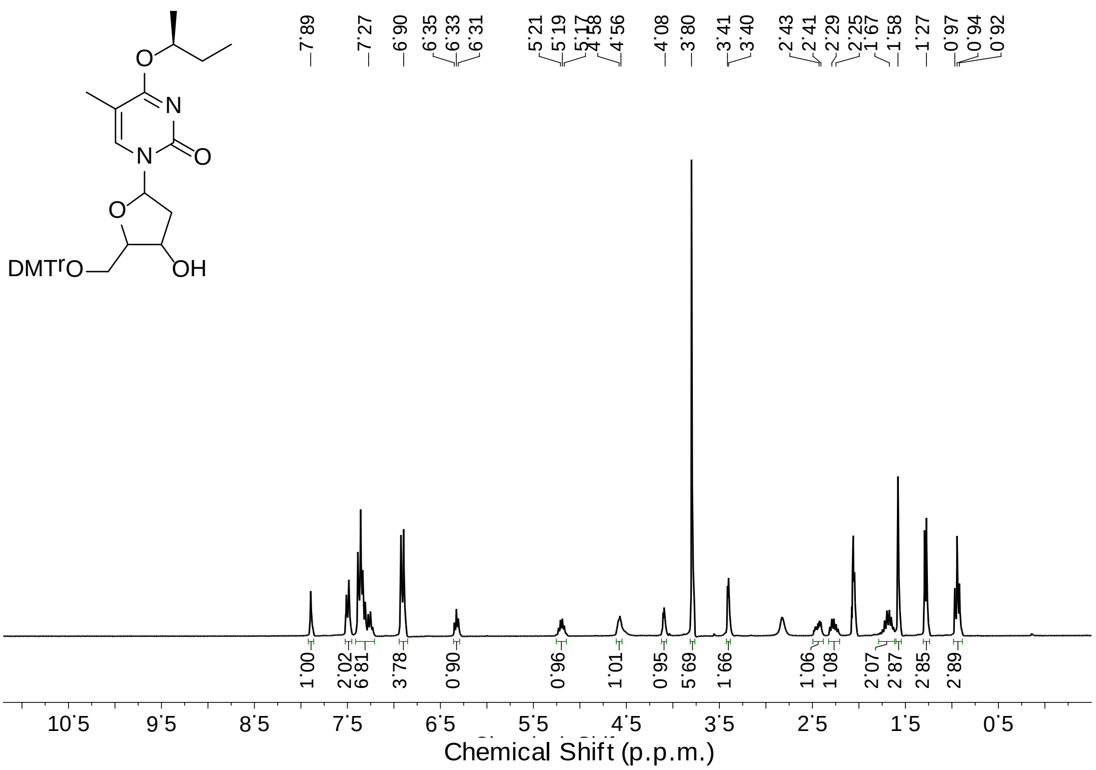

Figure S11. ^{1}H NMR spectrum of *O*^{4}-DMTr-(*S*)-*s*BudT (**3g**) (300 MHz, acetone-d_6, 25°C).

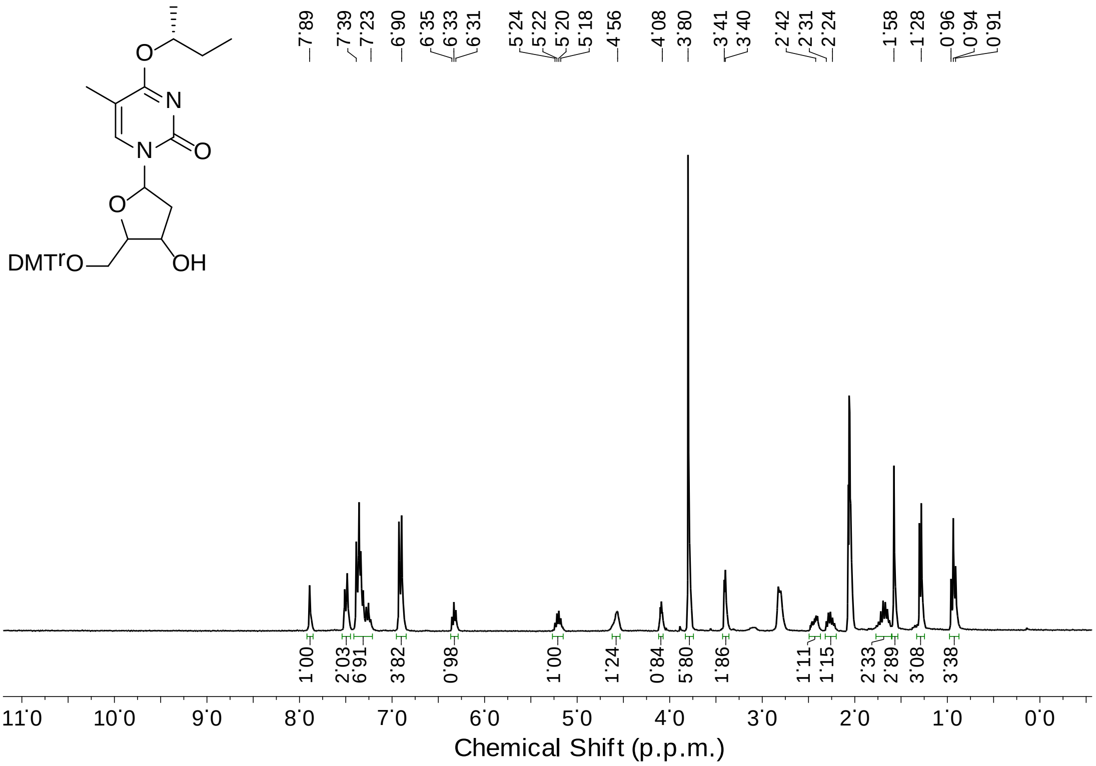

Figure S12. ^{1}H NMR spectrum of *O*^{4}-DMTr-(*R*)-*s*BudT (**3h**) (300 MHz, acetone-d_6, 25°C).

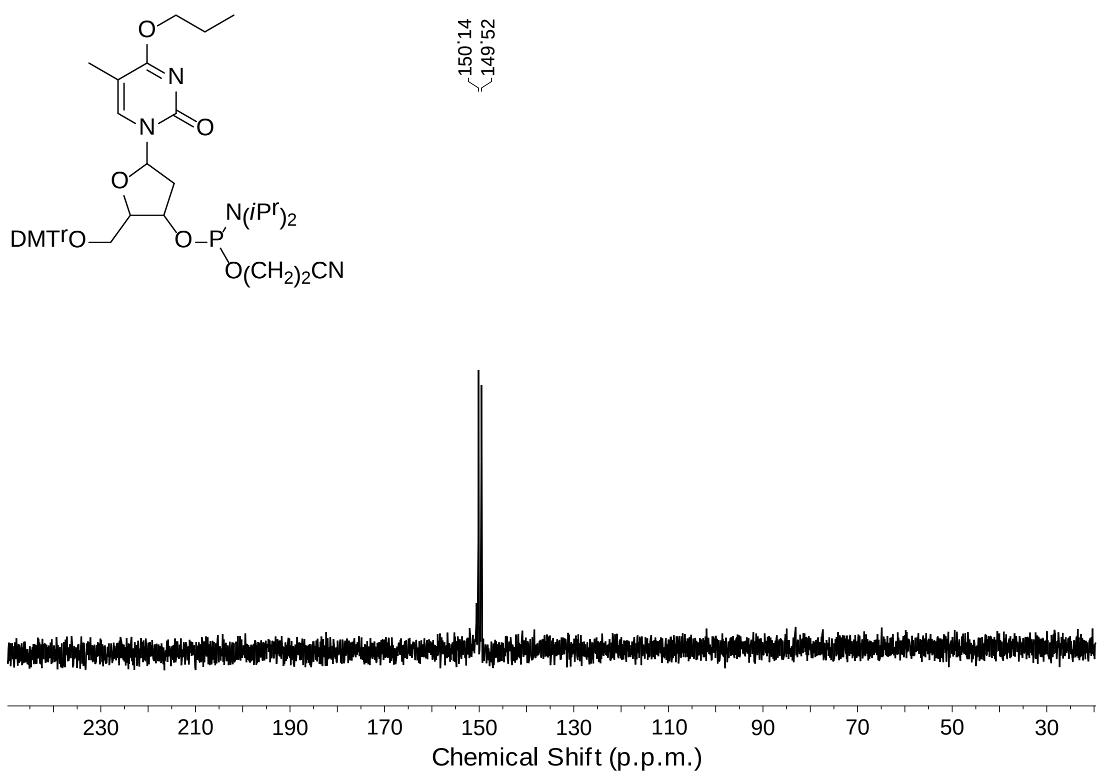

Figure S13. ^{31}P NMR spectrum of the phosphoramidite building block of *O*^{4}-*n*PrdT (**4c**) (80 MHz, $CDCl_3$, 25ºC).

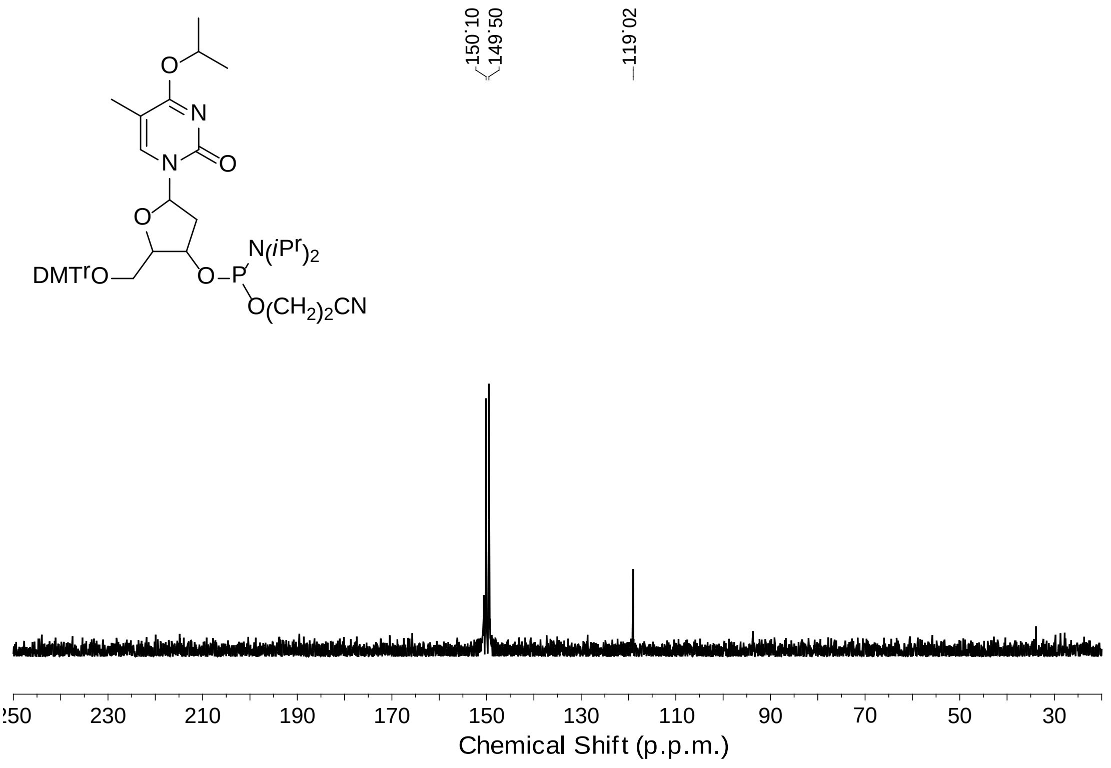

Figure S14. ^{31}P NMR spectrum of the phosphoramidite building block of *O*^{4}-*i*PrdT (**4d**) (80 MHz, $CDCl_3$, 25ºC).

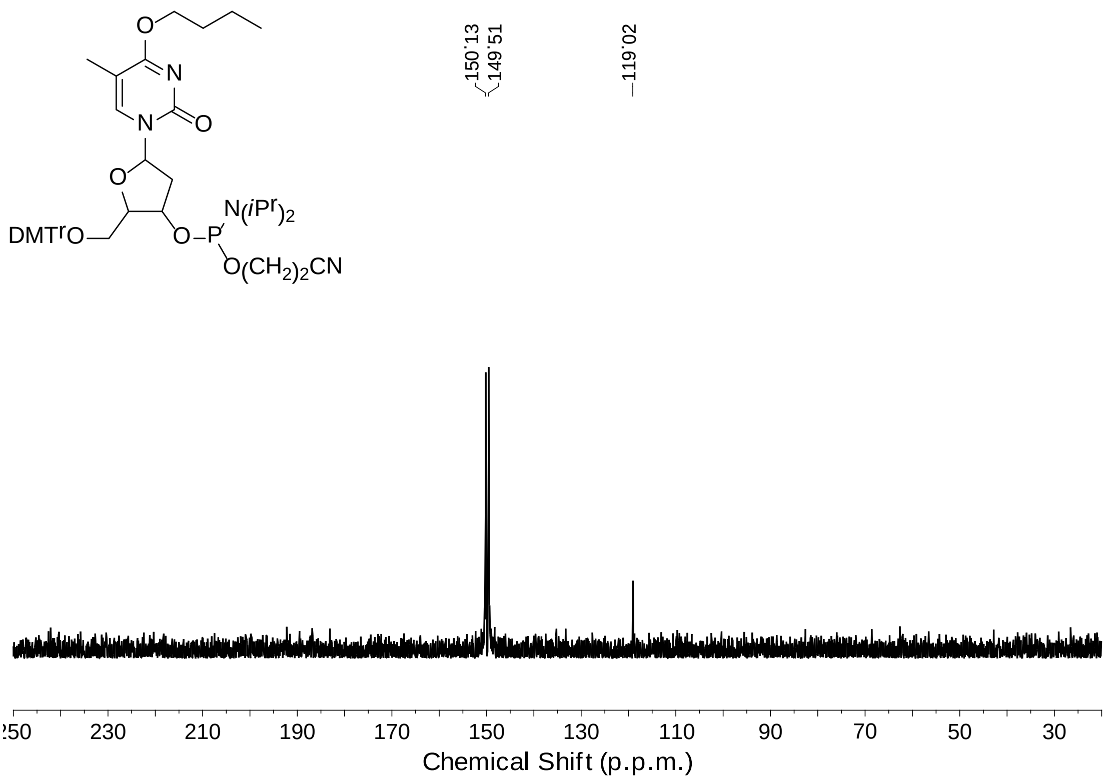

Figure S15. ^{31}P NMR spectrum of the phosphoramidite building block of *O*^{4}-*n*BudT (**4e**) (80 MHz, $CDCl_3$, 25ºC).

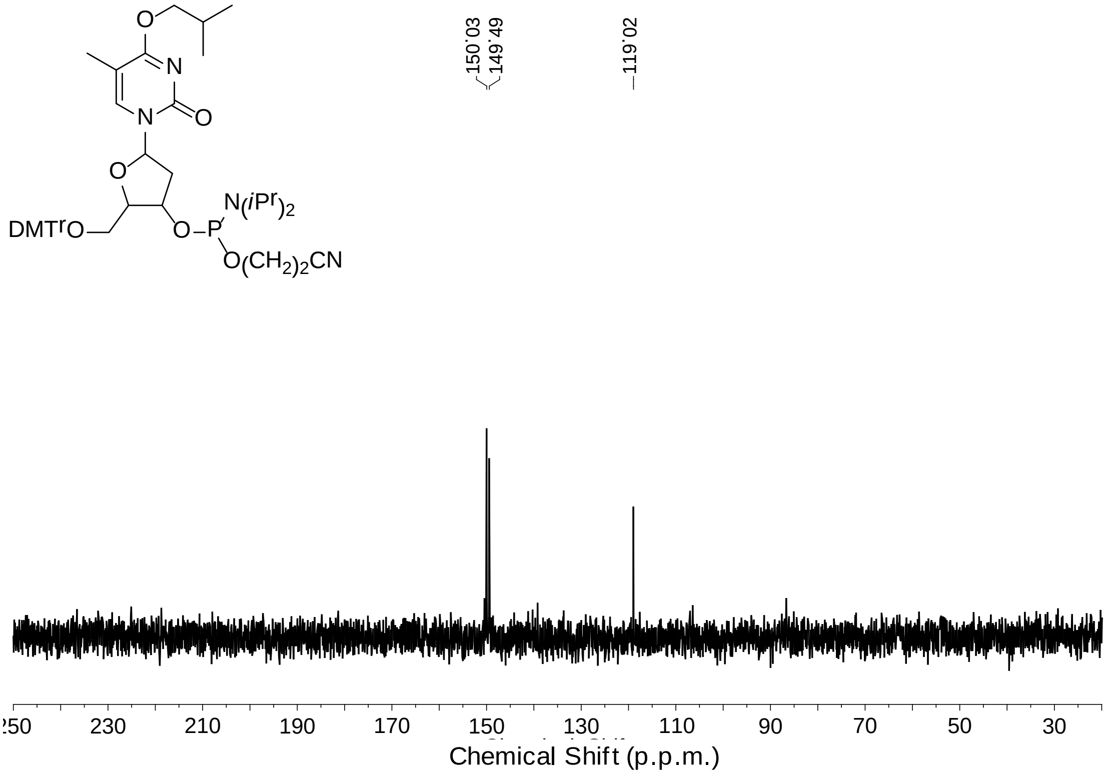

Figure S16. ^{31}P NMR spectrum of the phosphoramidite building block of *O*^{4}-*i*BudT (**4f**) (80 MHz, $CDCl_3$, 25ºC).

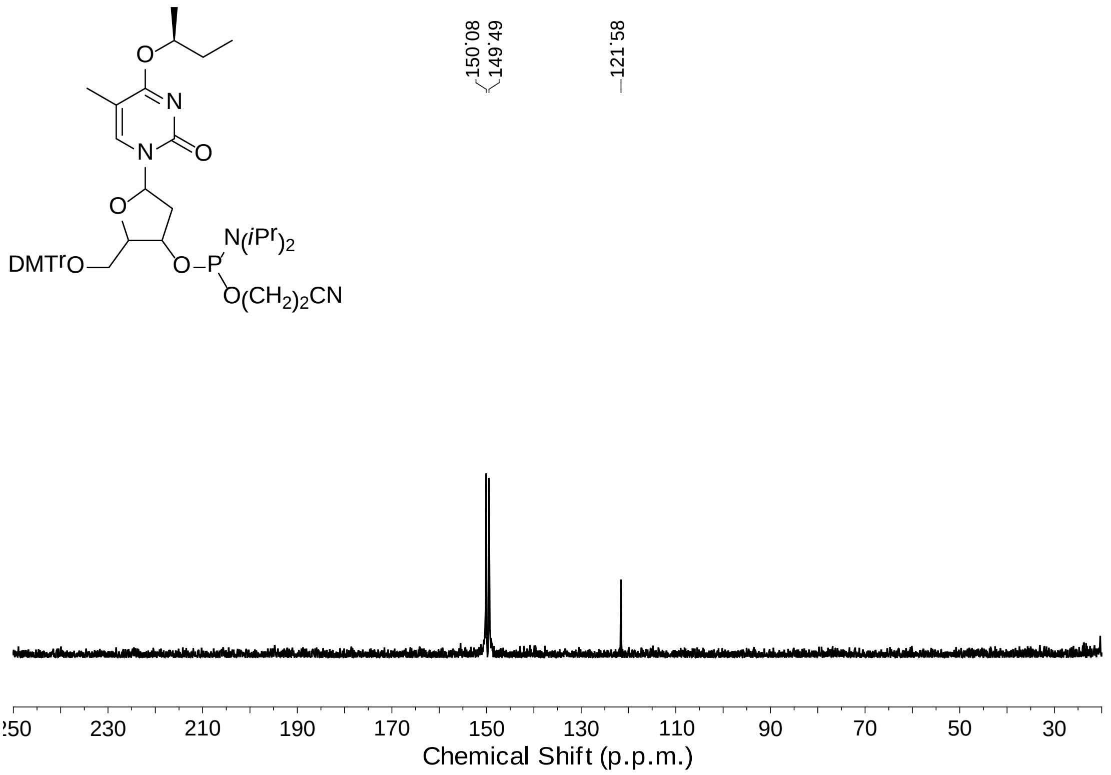

Figure S17. ^{31}P NMR spectrum of the phosphoramidite building block of *O*^{4}-(*S*)-*s*BudT (**4g**) (80 MHz, $CDCl_3$, 25°C).

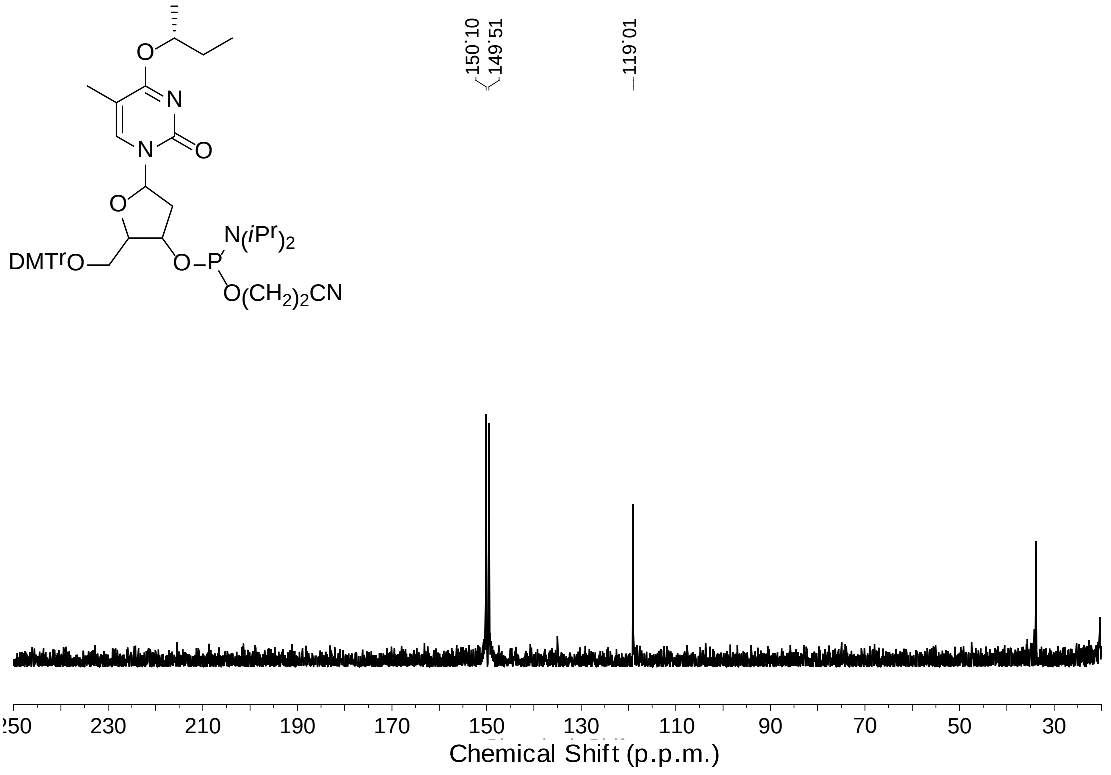

Figure S18. ^{31}P NMR spectrum of the phosphoramidite building block of *O*^{4}-(*R*)-*s*BudT (**4h**) (80 MHz, $CDCl_3$, 25°C).

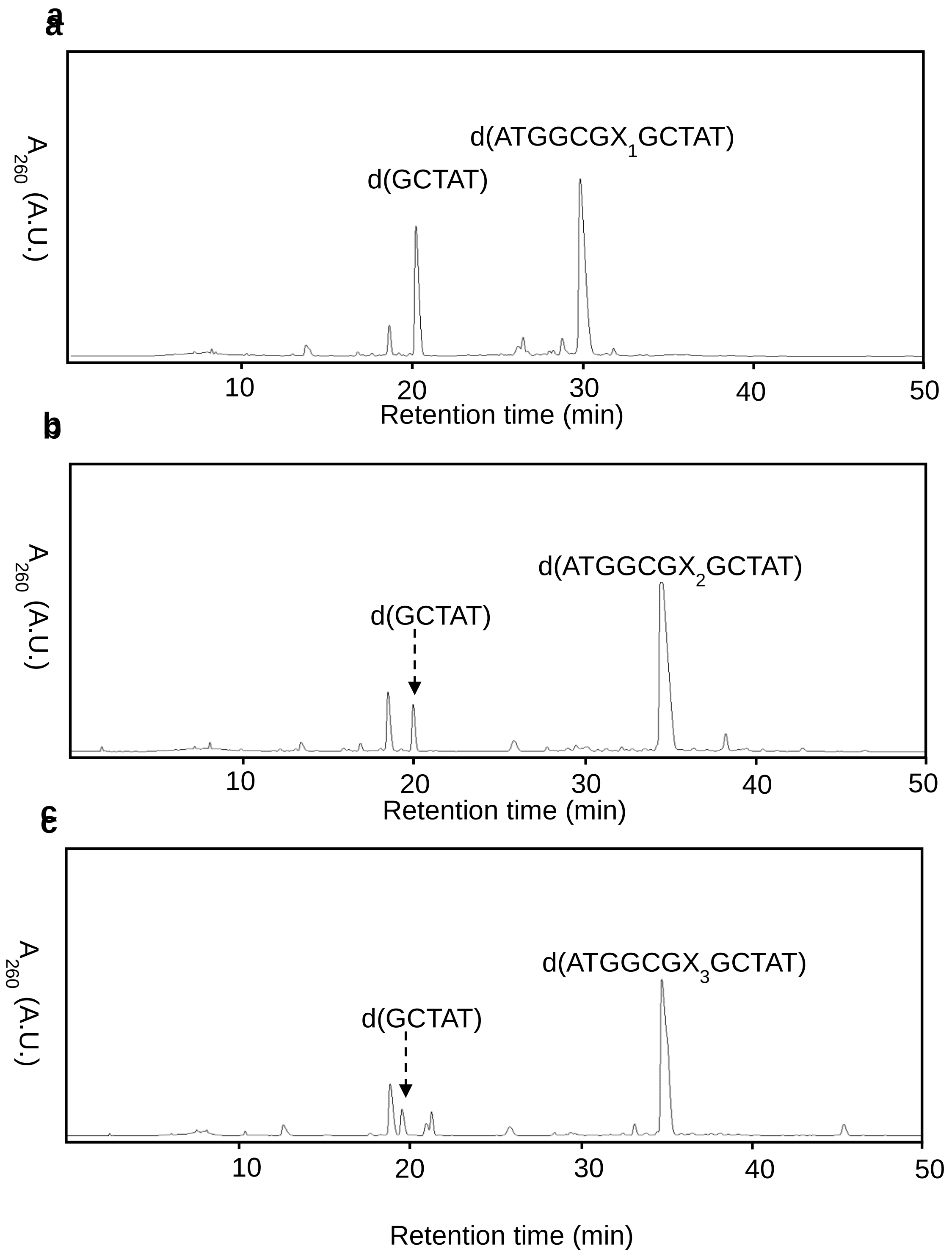
a
d(ATGGCGX1GCTAT)
d(GCTAT)
A260 (A.U.)
10
20
30
40
50
Retention time (min)
b
d(ATGGCGX2GCTAT)
d(GCTAT)
A260 (A.U.)
10
20
30
40
50
Retention time (min)
c
d(ATGGCGX3GCTAT)
d(GCTAT)
A260 (A.U.)
10
20
30
40
50
Retention time (min)

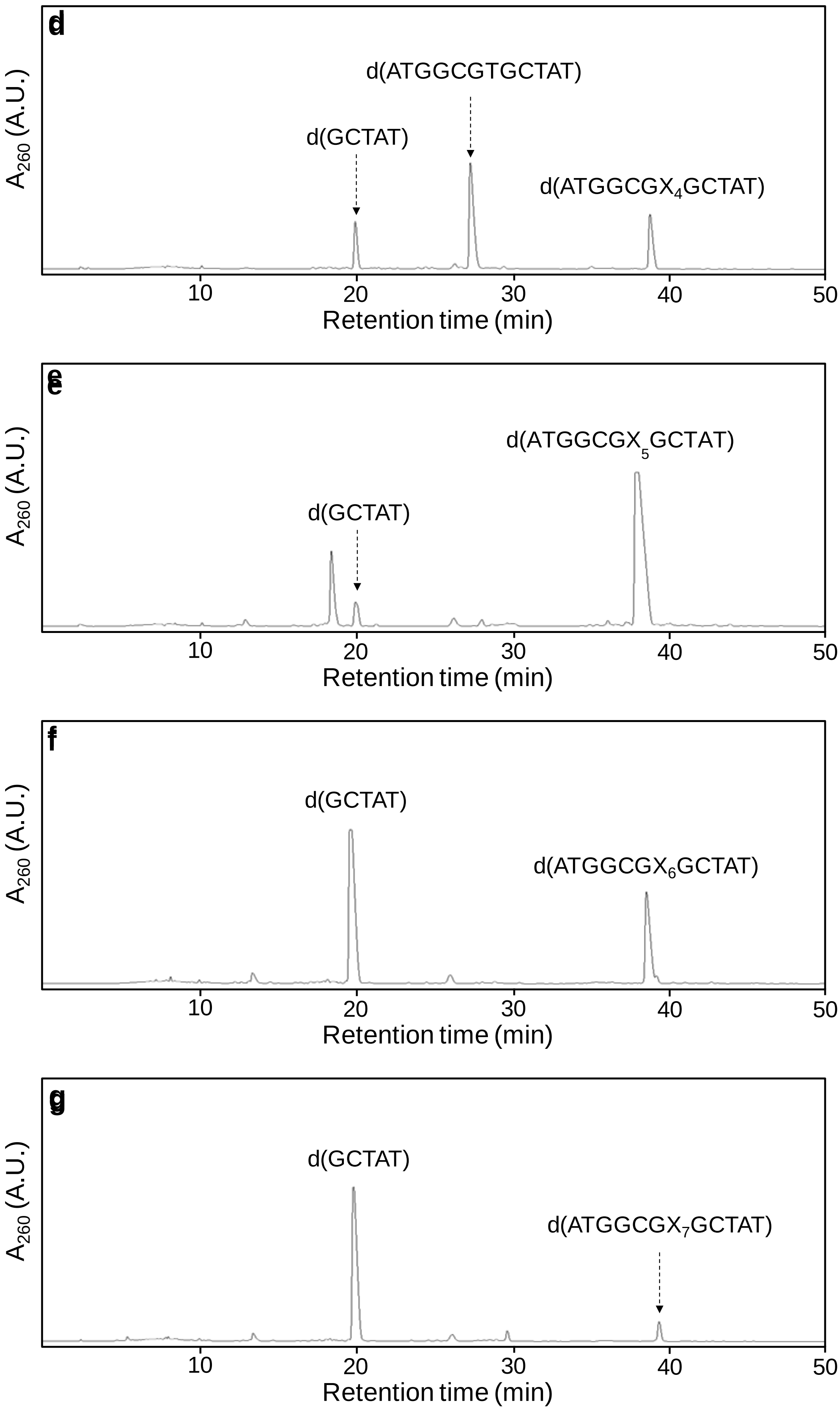
d
A260 (A.U.)
d(ATGGCGTGCTAT)
d(GCTAT)
d(ATGGCGX4GCTAT)
10 20 30 40 50
Retention time (min)
e
A260 (A.U.)
d(ATGGCGX5GCTAT)
d(GCTAT)
10 20 30 40 50
Retention time (min)
f
A260 (A.U.)
d(GCTAT)
d(ATGGCGX6GCTAT)
10 20 30 40 50
Retention time (min)
g
A260 (A.U.)
d(GCTAT)
d(ATGGCGX7GCTAT)
10 20 30 40 50
Retention time (min)

Figure S19. HPLC traces for the separations of the synthesized 12mer *O*[4]-alkyldT-bearing ODNs: (a) X_1= *O*[4]-MedT; (b) X_2 = *O*[4]-*n*PrdT; (c) X_3 = *O*[4]-*i*PrdT; (d) X_4 = *O*[4]-*n*BudT; (e) X_5 = *O*[4]-*i*BudT; (f) X_6 = *O*[4]-(*S*)-*s*BudT; (g) X_7 = *O*[4]-(*R*)-*s*BudT.

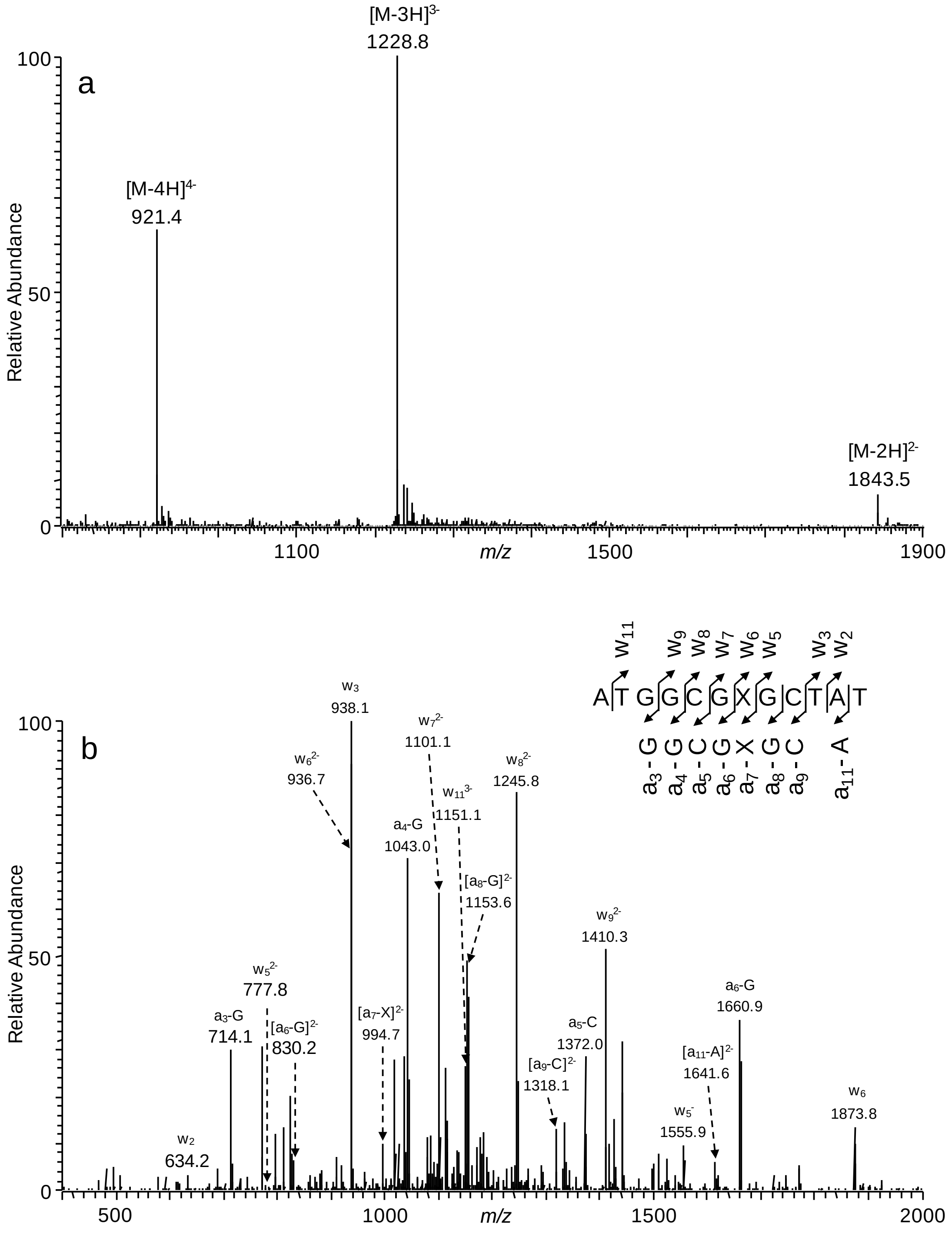

Figure S20. ESI-MS & MS/MS characterizations of d(ATGGCGXGCTAT), X=*O*[4]-MedT: (a) Negative-ion ESI-MS; (b) the product-ion spectrum of the $[M\text{-}3H]^{3-}$ ion (*m*/*z* 1228.8).

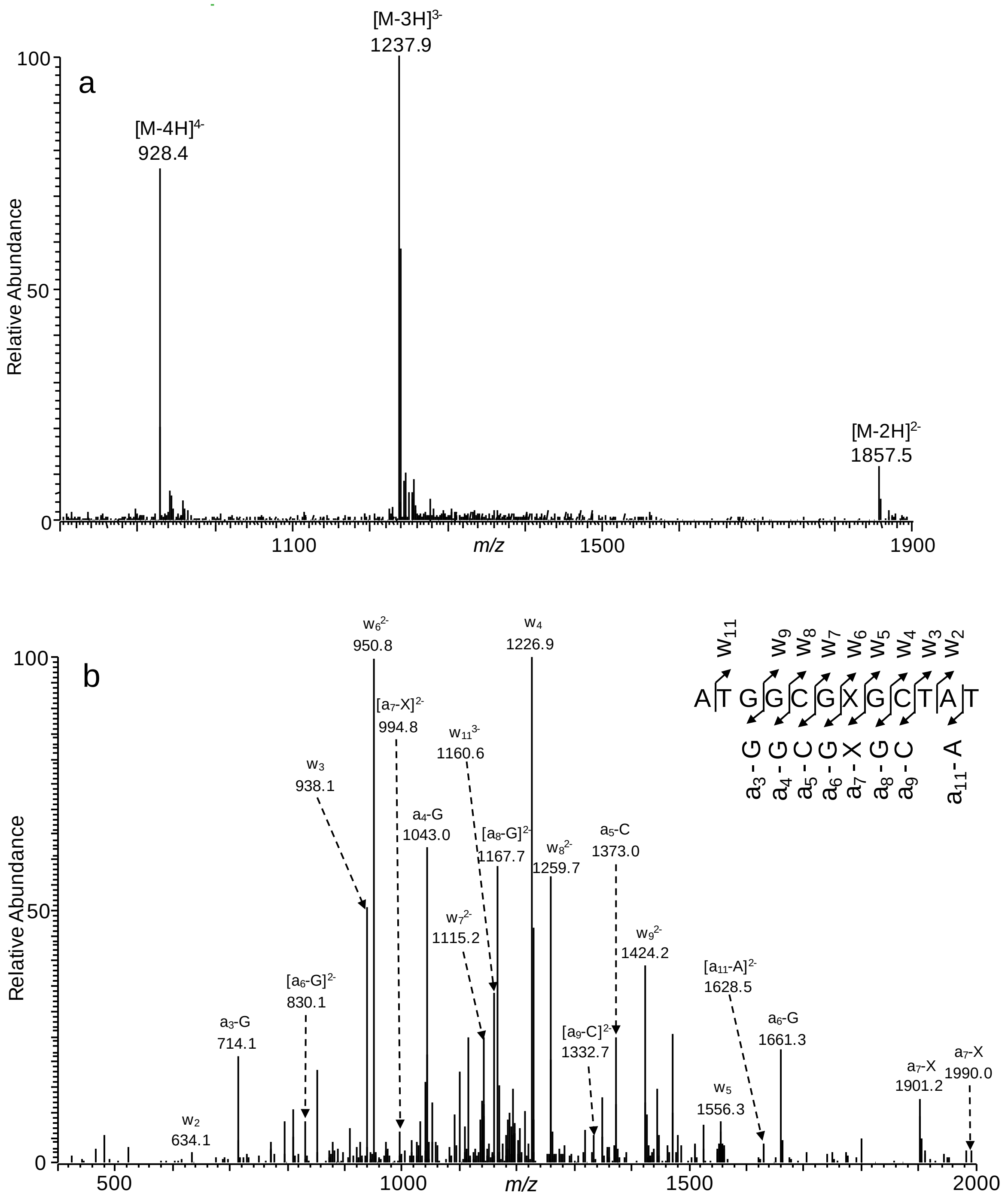

Figure S21. ESI-MS & MS/MS characterizations of d(ATGGCGXGCTAT), X=O^4-*n*PrdT: (a) Negative-ion ESI-MS; (b) the product-ion spectrum of the $[M-3H]^{3-}$ ion (*m*/*z* 1237.9).

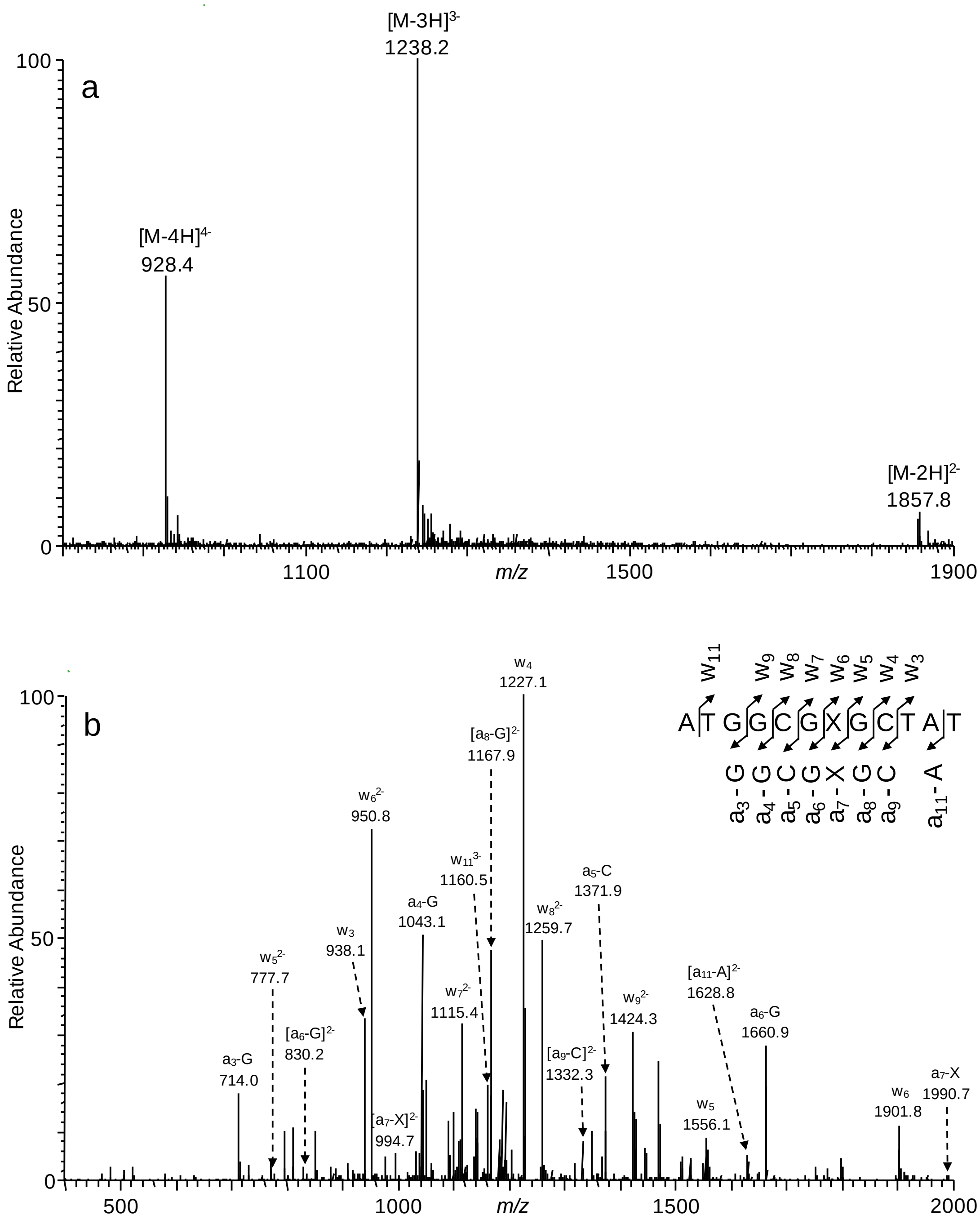

Figure S22. ESI-MS & MS/MS characterizations of d(ATGGCGXGCTAT), X=O^4-*i*PrdT: (a) Negative-ion ESI-MS; (b) the product-ion spectrum of the $[M-3H]^{3-}$ ion (*m*/*z* 1238.2).

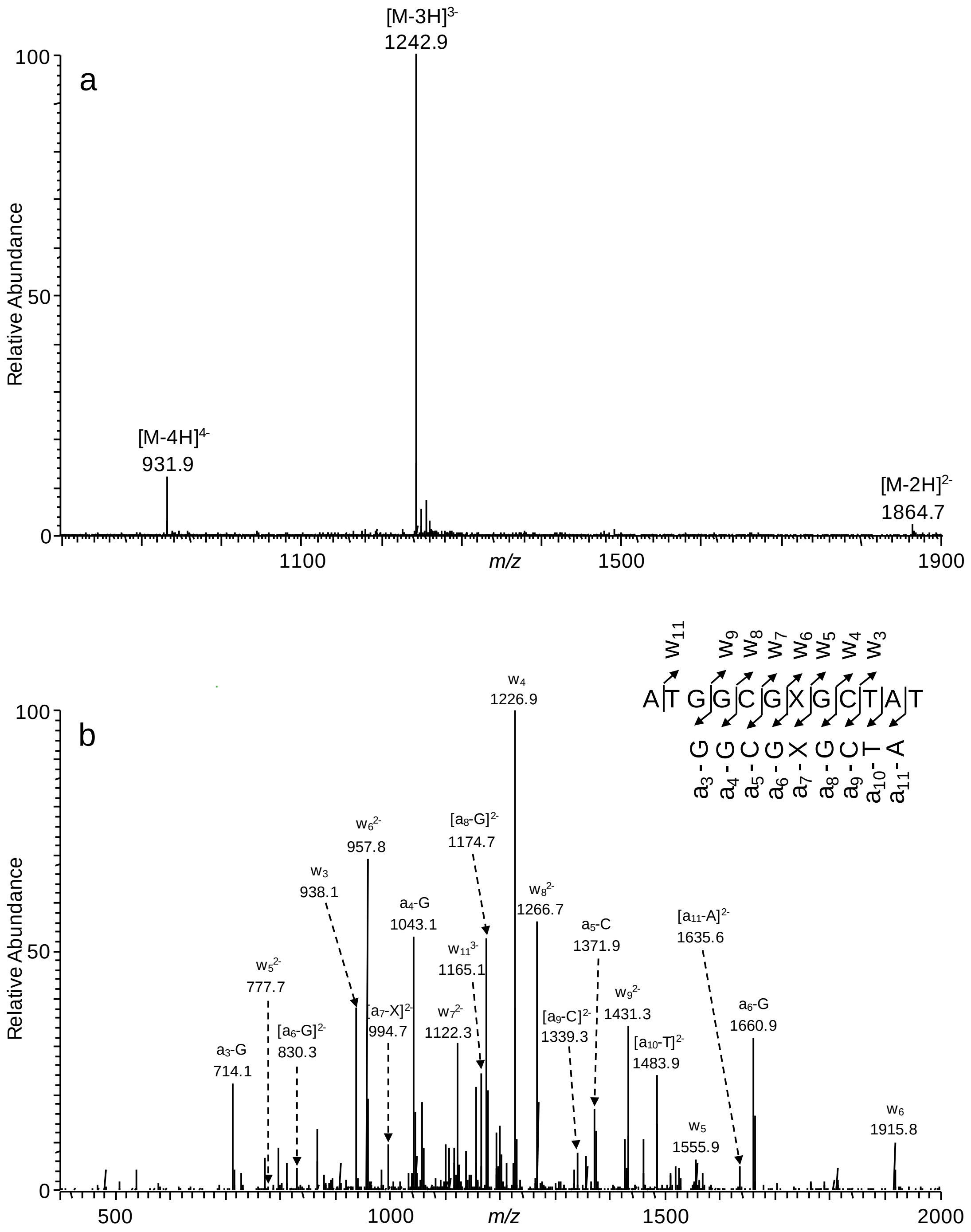

Figure S23. ESI-MS & MS/MS characterizations of d(ATGGCGXGCTAT), X=O^4-*n*BudT: (a) Negative-ion ESI-MS; (b) the product-ion spectrum of the $[M\text{-}3H]^{3-}$ ion (*m/z* 1242.9).

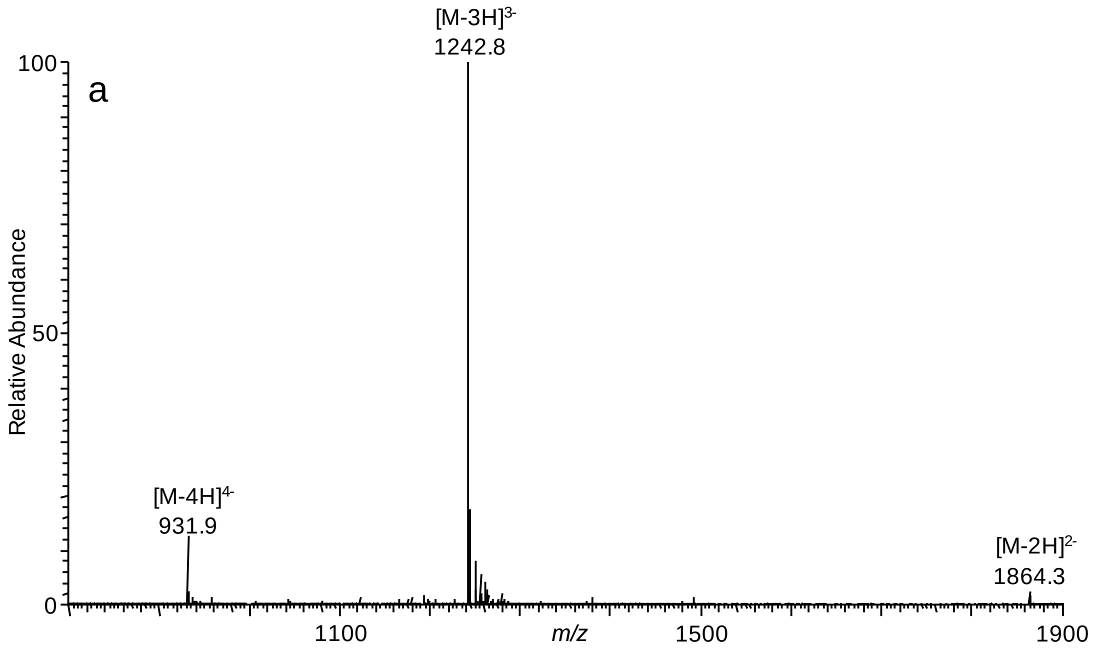

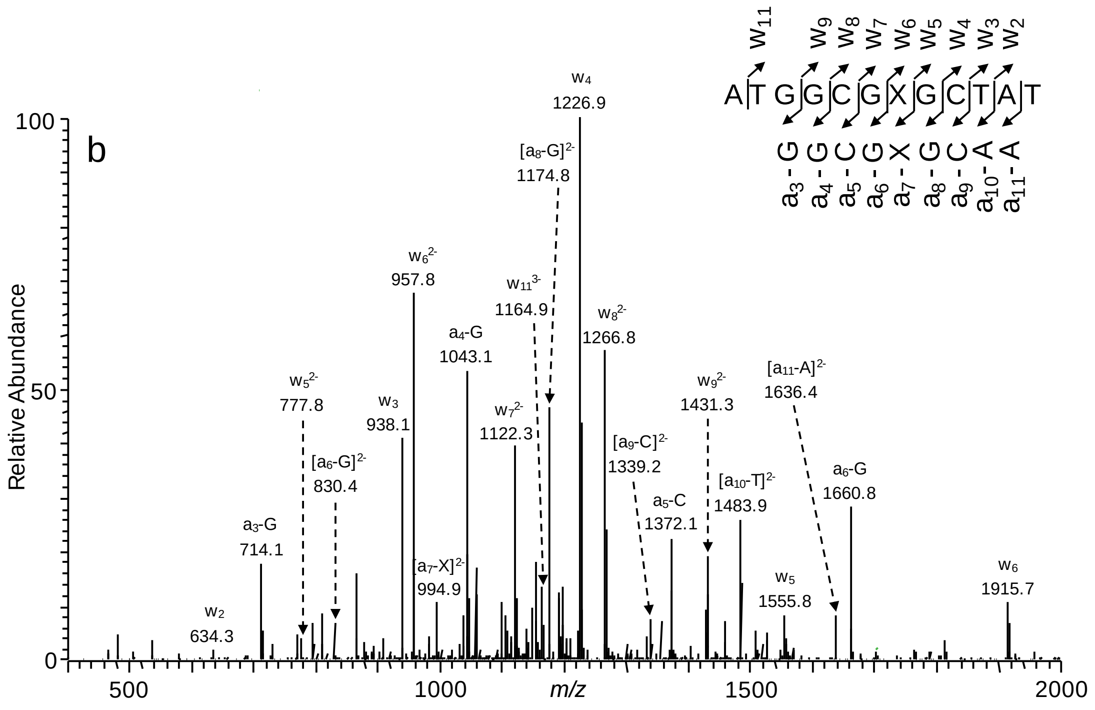

Figure S24. ESI-MS & MS/MS characterizations of d(ATGGCGXGCTAT), X=O^4-*i*BudT: (a) Negative-ion ESI-MS; (b) the product-ion spectrum of the $[M\text{-}3H]^{3-}$ ion (*m/z* 1242.8).

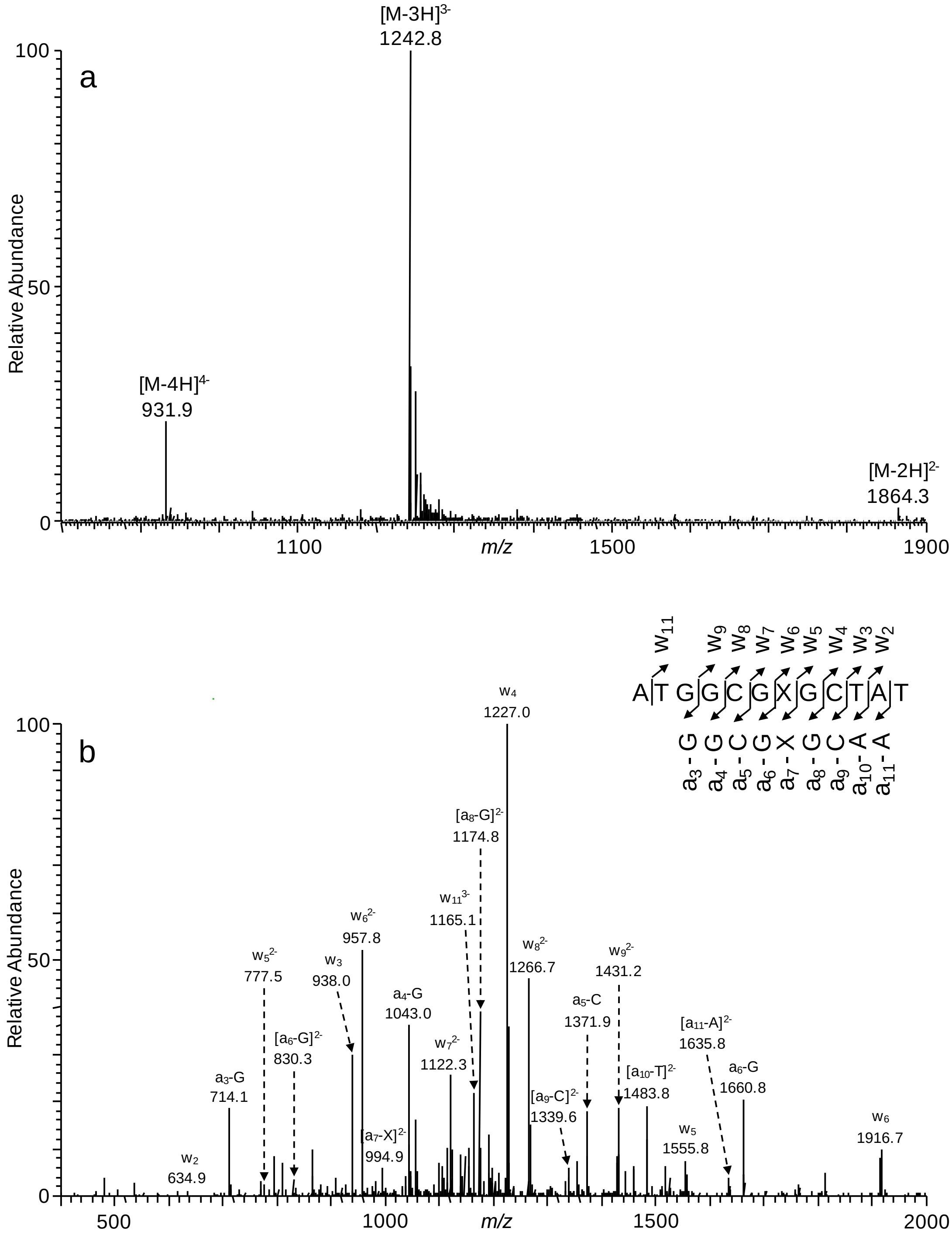

Figure S25. ESI-MS & MS/MS characterizations of d(ATGGCGXGCTAT), X=*O*^{4}-(*S*)-*s*BudT: (a) Negative-ion ESI-MS; (b) the product-ion spectrum of the [M-3H]$^{3-}$ ion (*m*/*z* 1242.8).

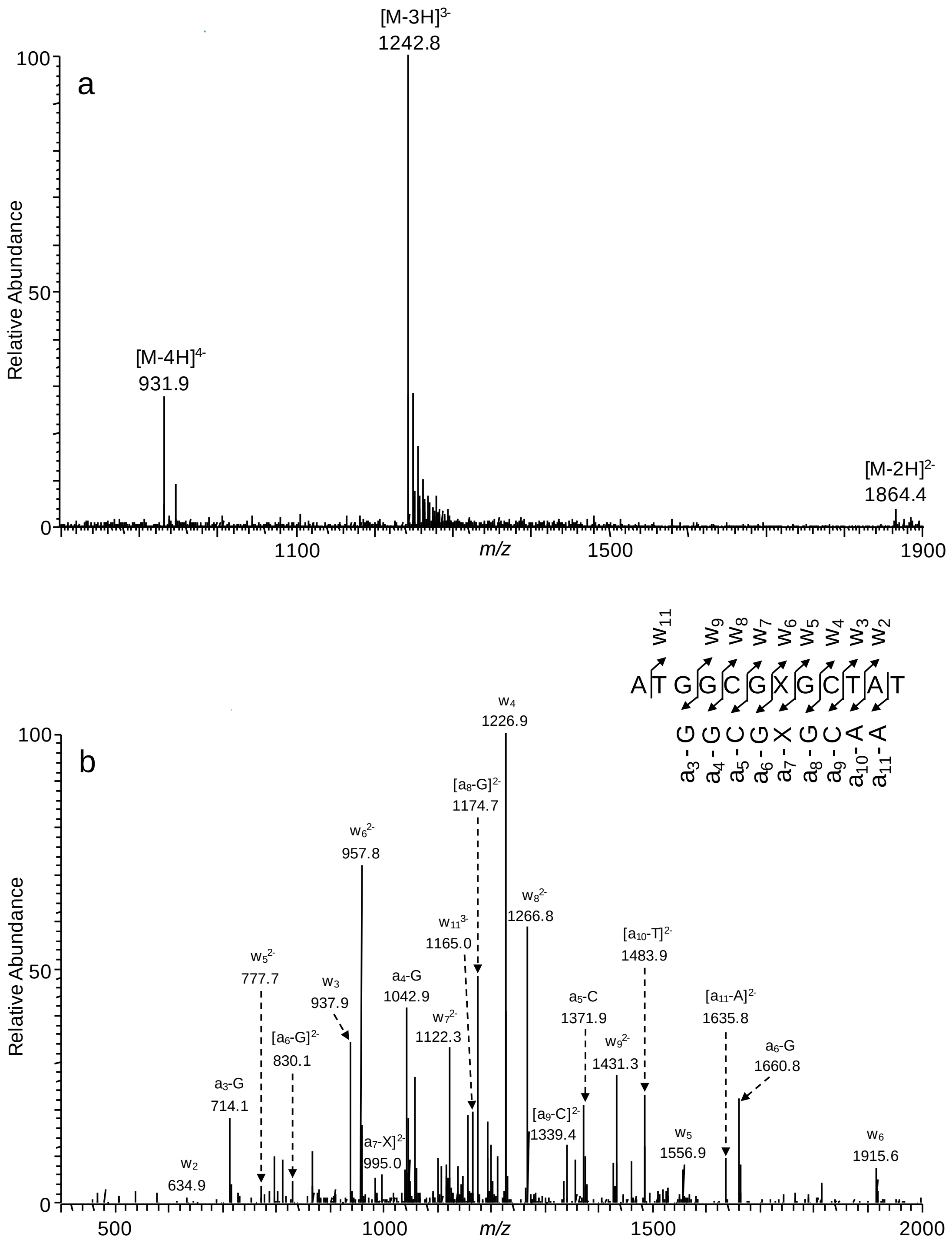

Figure S26. ESI-MS & MS/MS characterizations of d(ATGGCGXGCTAT), X=O^4-(*R*)-*s*BudT (a) Negative-ion ESI-MS; (b) the product-ion spectrum of the $[M\text{-}3H]^{3-}$ ion (*m*/*z* 1242.8).

a. Construction of Lesion-containing genome:

5'-CAGGAAAGCTATGACCATGATTCAGTGAGTGGAAGACATGGCGXGCTATAATTCACTGGCCGTCGTTTTACAACGTCGTGACTGGGAA-3′

3'-CTTTCGATACTGGTACTAAGTCACTCACCTTC-5′   3′-CGATATTAAGTGACCGGCAGCAAAA-5′

b. Construction of competitor genome:

5′-CAGGAAAGCTATGACCATGATTCAGTGAGTGGAAGACATGGCGATAAGCTATAATTCACTGGCCGTCGTTTTACAACGTCGTGACTGGGAA-3′

3′-CTTTCGATACTGGTACTAAGTCACTCACCTTC-5′   3′-CGATATTAAGTGACCGGCAGCAAAA-5′

Figure S27. Schematic diagrams showing the construction of the lesion-containing (a) and competitor genomes. Displayed are the partial sequence of the linearized M13, the 22-mer lesion-containing insert or the 25-mer lesion-free insert (shown in red), and the two scaffolds employed for the ligation reactions. The lesion site is underlined (X).

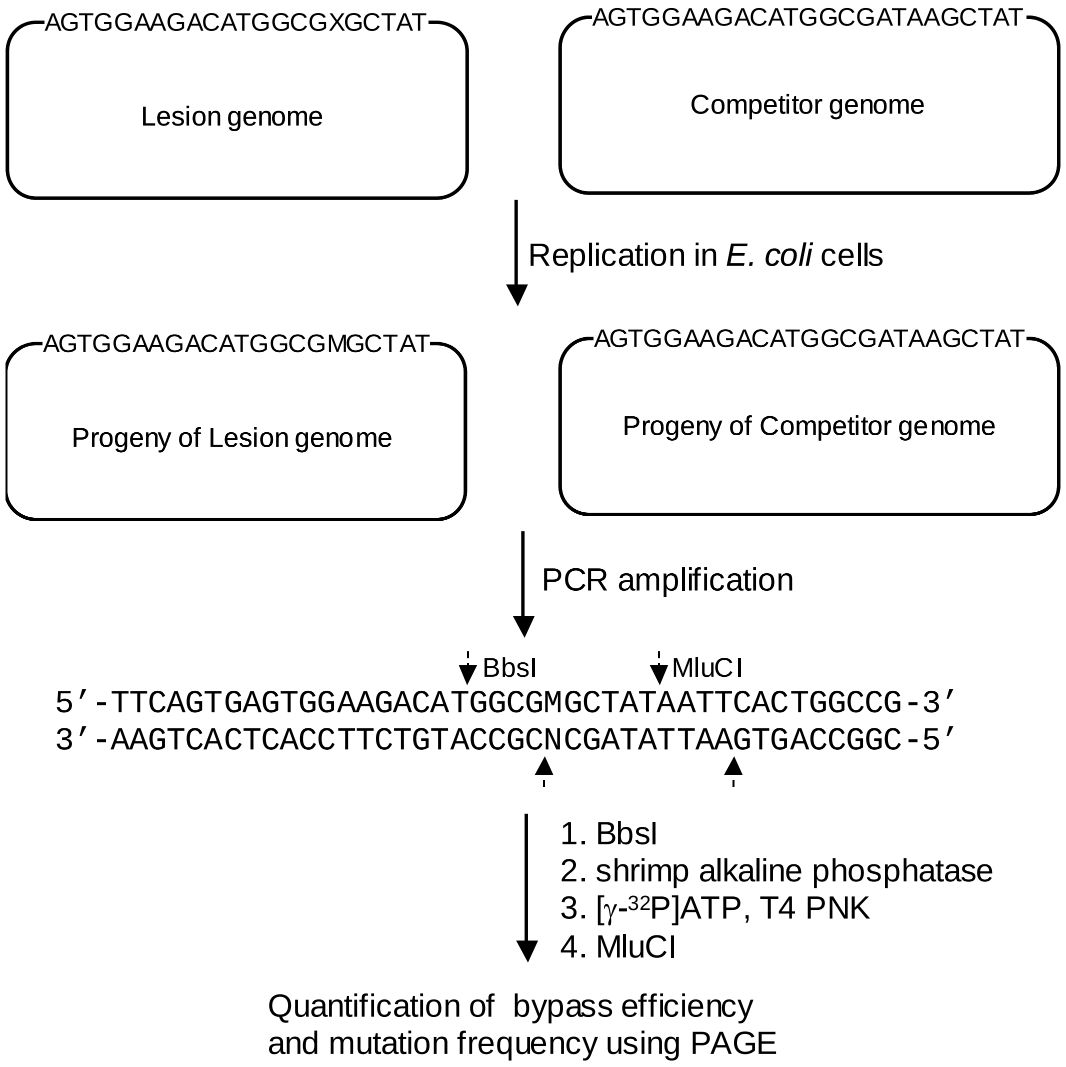

Figure S28. Restriction digestion and post-labeling method for determining the bypass efficiency and mutation frequency of O^4-alkyldT in *E. coli* cells. 'X' in the 22-mer DNA strand designates dT or O^4-alkyldT. BbsI and MluCI restriction endonuclease digestion sites are indicated with broken arrows. Only partial sequences of the PCR products for the lesion-containing genome are shown, and the PCR products of the competitor genome are not shown. The order for the two restriction enzyme digestion was also reversed to selectively radiolabel the complementary strand and to better resolve the product with T→C mutation from other restriction fragments (See Materials and Methods). For LC-MS/MS analysis, the PCR products were digested with BbsI and MluCI prior to digestion using shrimp alkaline phosphatase, where the [^{32}P]-labeling step involving the use of T4 polynucleotide kinase (T4 PNK) was omitted.

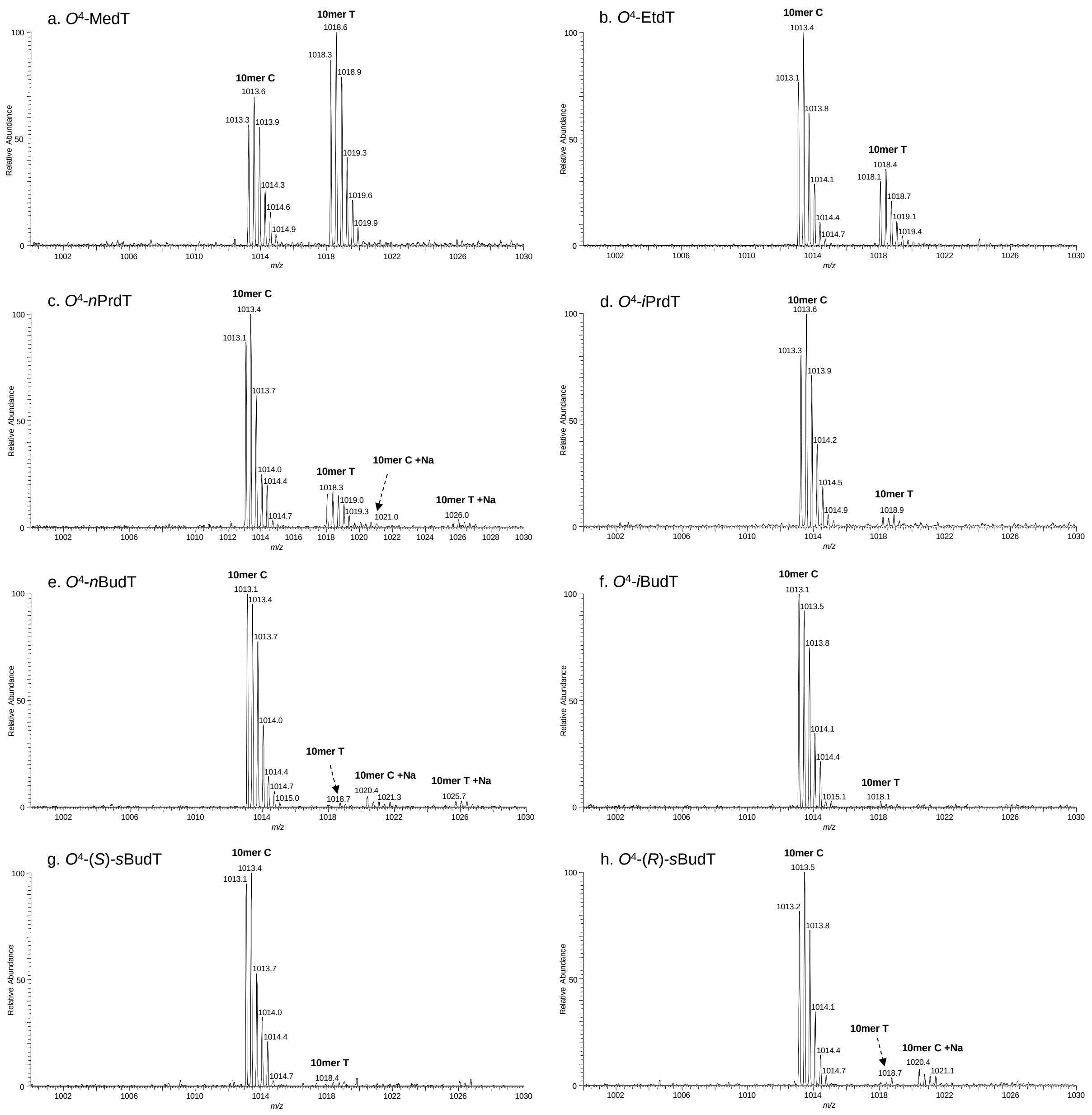

Figure S29. Higher-resolution "ultra-zoom scan" ESI-MS of the restriction fragments for the PCR products from the replication of (a) *O*4-MedT, (b) *O*4-EtdT, (c) *O*4-*n*PrdT, (d) *O*4-*i*PrdT, (e) *O*4-*n*BudT, (f) *O*4-*i*BudT, (g) *O*4-(*S*)-*s*BudT, (h) *O*4-(*R*)-*s*BudT bearing single-stranded M13 genomes in SOS-induced wild-type AB1157 cells. Displayed in (a)-(h) are the $[M - 3H]^{3-}$ ions for the lesion-containing strand products. All the mutagenic products were further confirmed by MS/MS analyses, and representative MS/MS results for the restriction fragments corresponding to replication products for *O*4-MedT are shown in Figures S30. To avoid the ambiguity introduced by Na^+ adduction, restriction products from complementary strand were also interrogated by MS and MS/MS (Figures S31-S32).

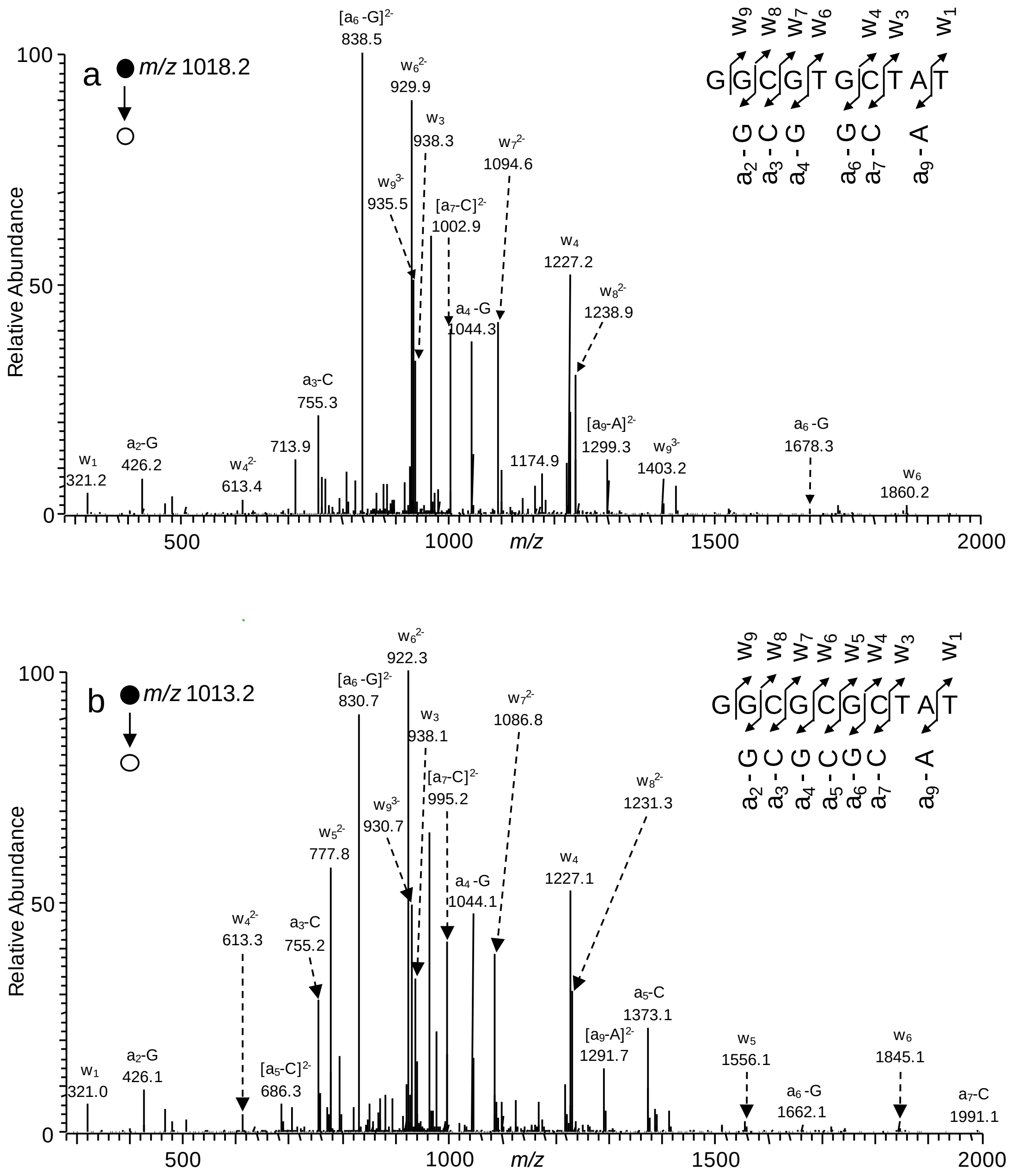

Figure S30. LC-MS and MS/MS for the identification of restriction fragments of PCR products. MS/MS for the $[M – 3H]^{3-}$ ions of (a) 10 mer T (non-mutagenic product), and (b) 10 mer C (T→C mutation). The T→C mutation was also monitored using the complementary strand (See Figures S31-S32).

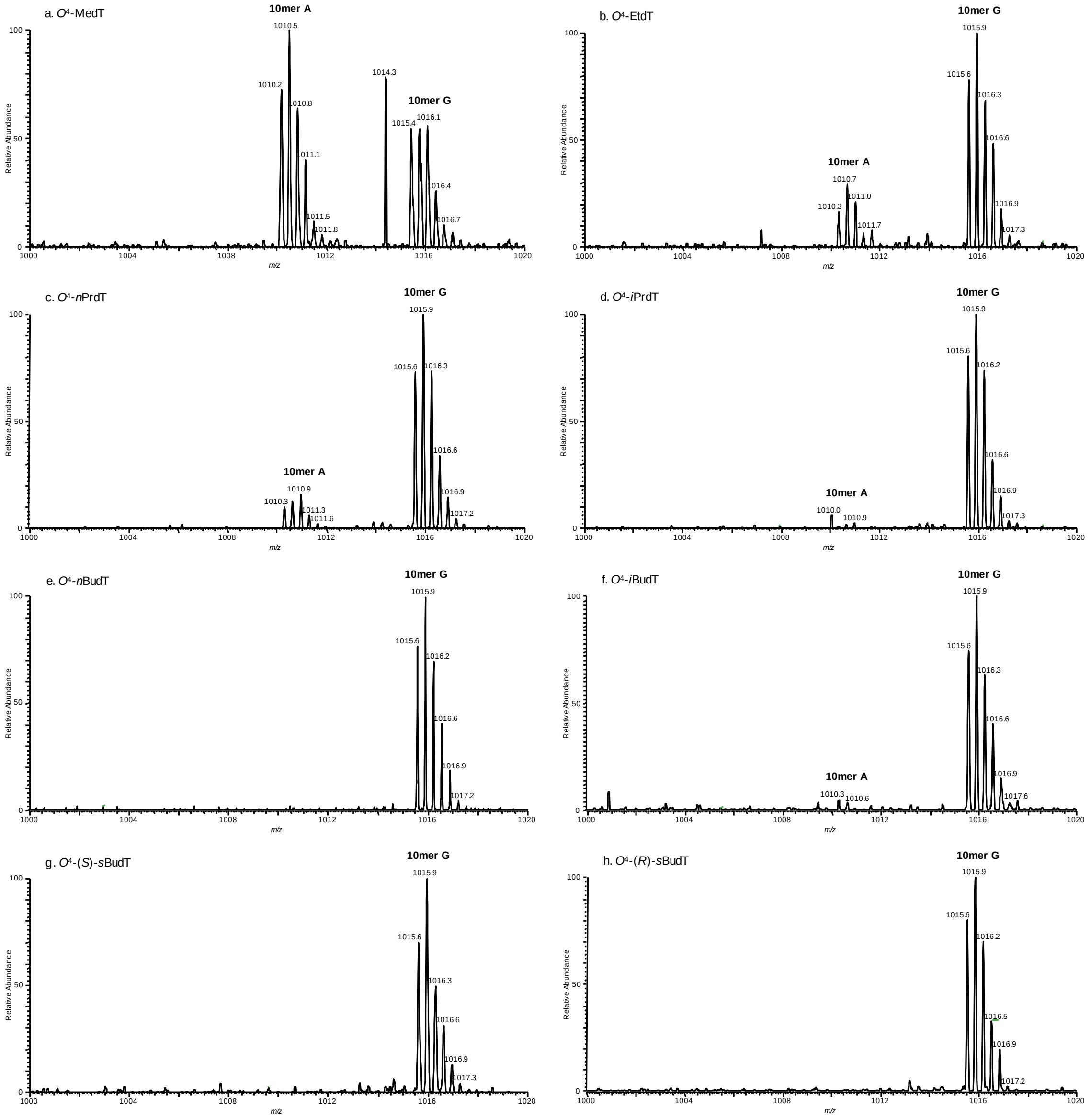

Figure S31. Higher-resolution "ultra-zoom scan" ESI-MS for the restriction fragments for the PCR products from the replication of (a) *O*^{4}-MedT, (b) *O*^{4}-EtdT, (c) *O*^{4}-*n*PrdT, (d) *O*^{4}-*i*PrdT, (e) *O*^{4}-*n*BudT, (f) *O*^{4}-*i*BudT, (g) *O*^{4}-(*S*)-*s*BudT, (h) *O*^{4}-(*R*)-*s*BudT bearing single-stranded M13 genomes in SOS-induced wild-type AB1157 cells. Displayed in (a)-(h) are the $[M – 3H]^{3-}$ ions for the lesion complementary strand products. All the mutagenic products were further confirmed by MS/MS analyses, and representative MS/MS results for *O*^{4}-MedT are shown in Figures S32. To avoid the ambiguity introduced by Na^+ adduction, restriction products from complementary strand were also interrogated by MS and MS/MS (Figures S29-S30).

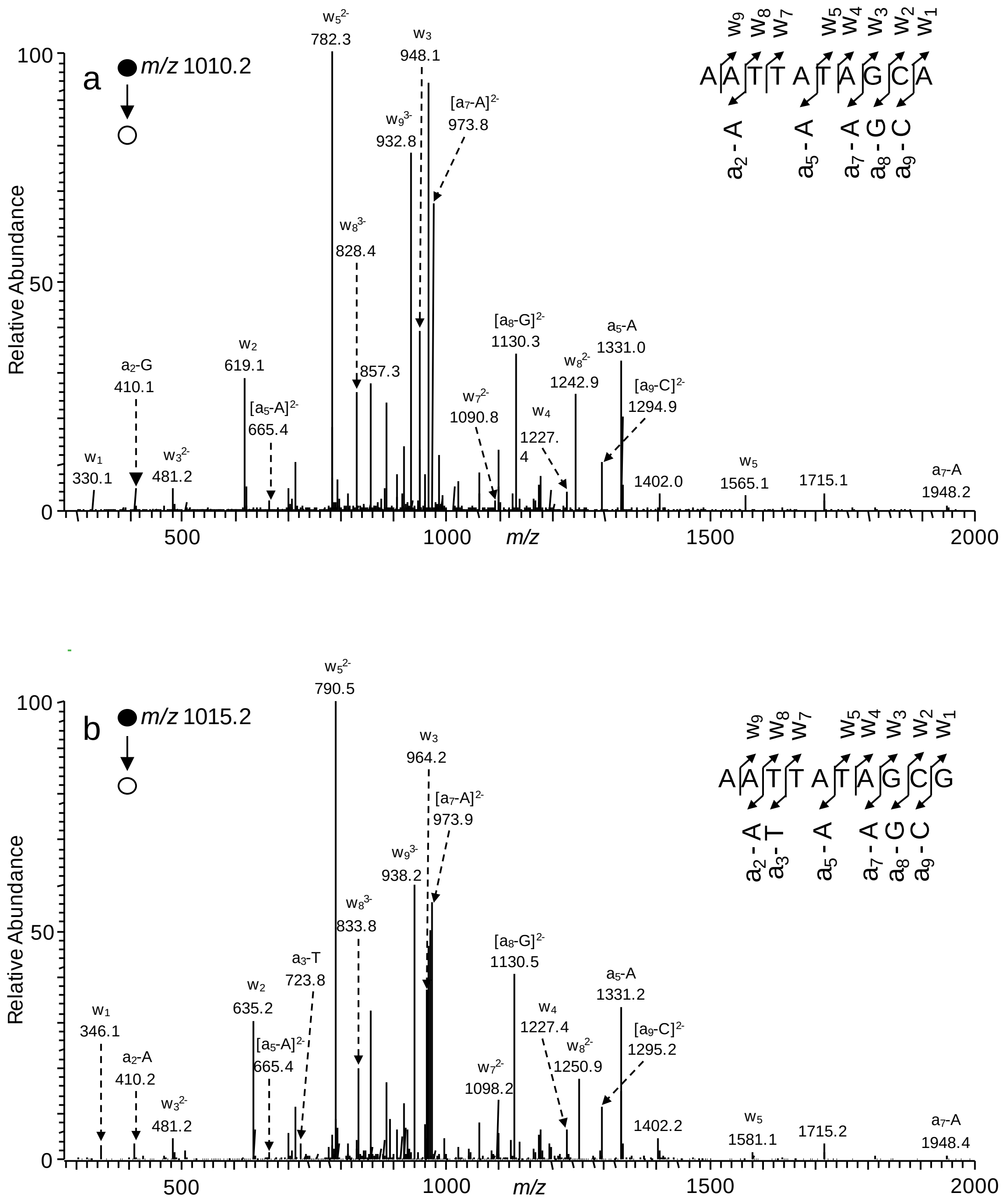

Figure S32. LC-MS and MS/MS for the identification of restriction fragments of PCR products. MS/MS for the $[M – 3H]^{3-}$ ions of (a) 10 mer A (non-mutagenic product), and (b) 10 mer G (corresponding to T → C mutation at the lesion site). The T→C mutation was also monitored using the lesion-situated strand (See Figures S29-S30).

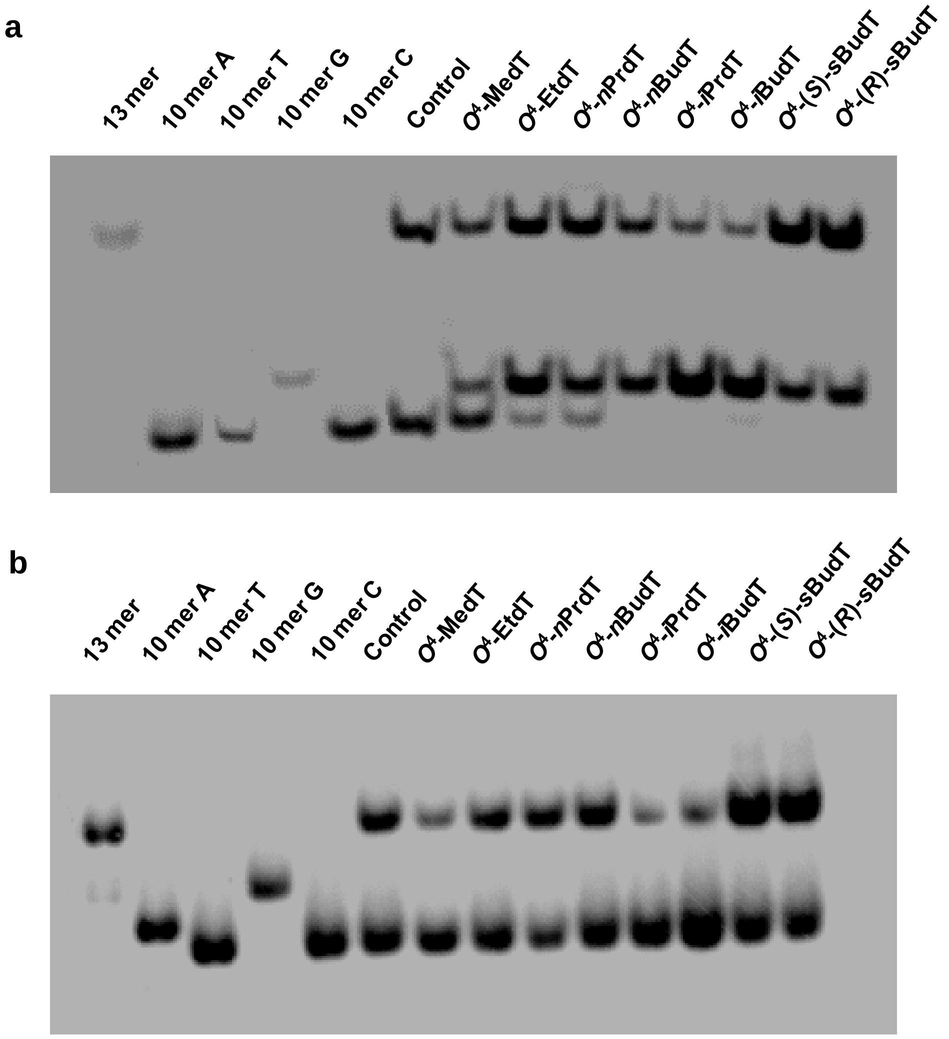

Figure S33. Native PAGE (30%) for monitoring the bypass efficiencies and mutation frequencies of O^4-alkylated-dT in wild-type (WT) AB1157 *E. coli* cells. (a-b) Gel images showing the 13-mer and 10-mer products released from the bottom-strand (opposite to lesion-containing strand) of the PCR products of the progeny of the competitor genome and the control or lesion-carrying genome, where 10mer A, 10mer C, 10mer G, and 10mer T represent the [5′-^{32}P]-labeled standard ODNs 5′-AATTATAGCN-3′, with ‘N’ being A, C, G, and T, respectively. (c) Gel image showing the 13-mer and 10-mer products released from the top-strand (lesion-containing strand) of the PCR products of the progeny of the competitor genome and the control or lesion-carrying genome, where 10mer A, 10mer C, 10mer G, and 10mer T represent the [5′-^{32}P]-labeled standard ODNs 5′-GGCGMGCTAT-3′, with ‘M’ being A, C, G, and T, respectively.

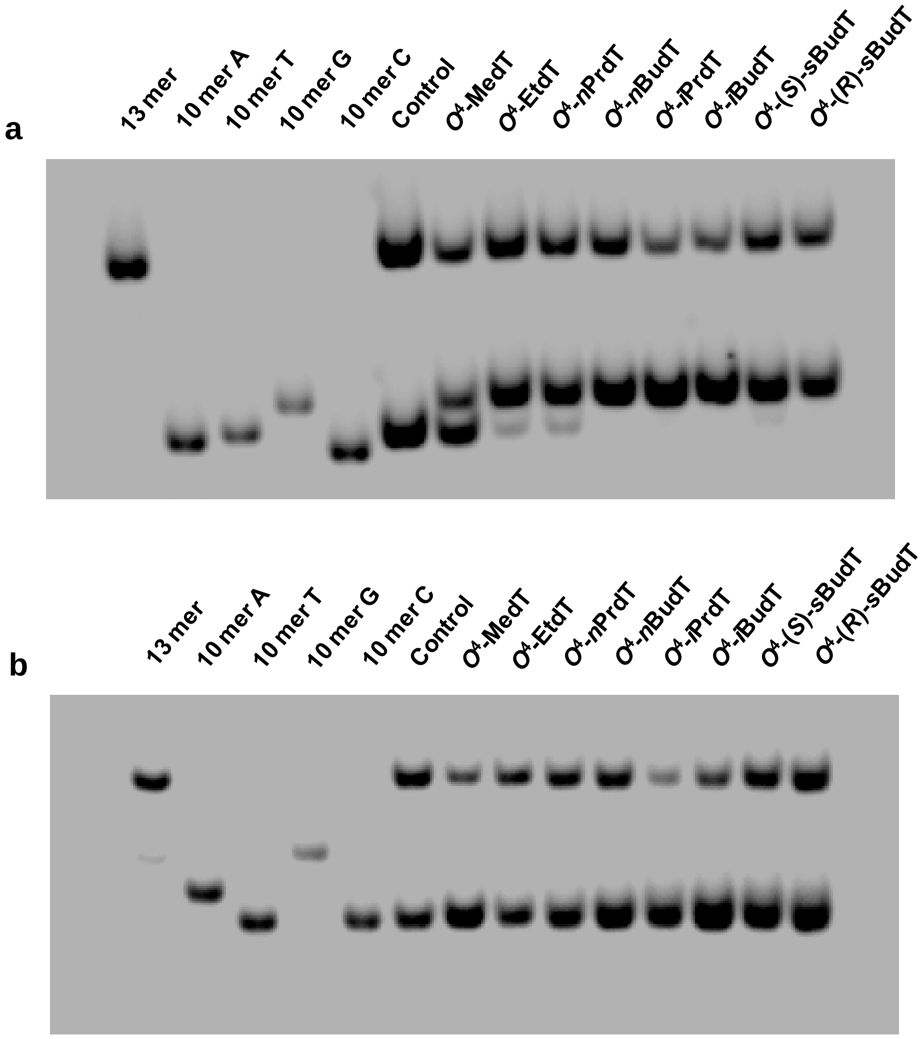

Figure S34. Native PAGE (30%) for monitoring the bypass efficiencies and mutation frequencies of O^4-alkylated-dT in SOS-induced AB1157 *E. coli* cells deficient in Pol II. (a) Gel image showing the 13-mer and 10-mer products released from the bottom-strand (opposite to lesion-containing strand) of the PCR products of the progeny of the competitor genome and the control or lesion-carrying genome, where 10mer A, 10mer C, 10mer G, and 10mer T represent the [5′-^{32}P]-labeled standard ODNs 5′-AATTATAGCN-3′, with 'N' being A, C, G, and T, respectively. (b) Gel image showing the 13-mer and 10-mer products released from the top-strand (lesion-containing strand) of the PCR products of the progeny of the competitor genome and the control or lesion-carrying genome, where 10mer A, 10mer C, 10mer G, and 10mer T represent the [5′-^{32}P]-labeled standard ODNs 5′-GGCGMGCTAT-3′, with 'M' being A, C, G, and T, respectively.

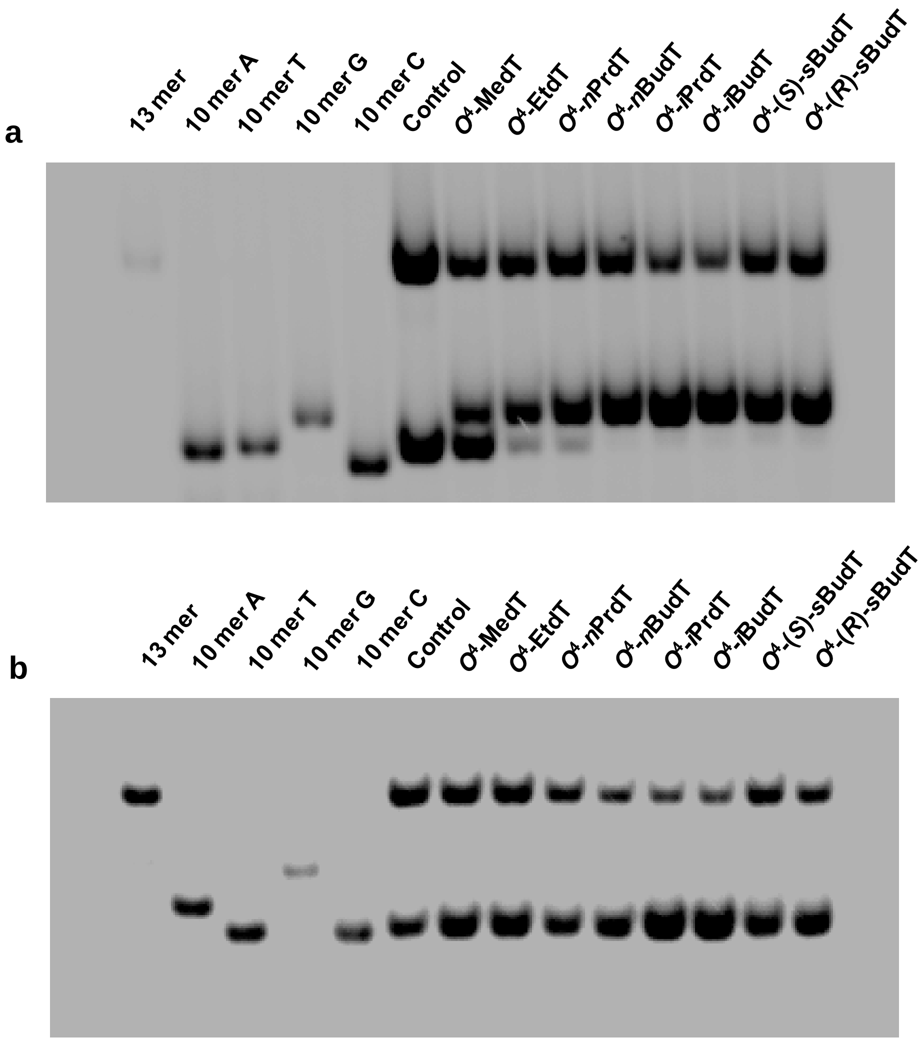

Figure S35. Native PAGE (30%) for monitoring the bypass efficiencies and mutation frequencies of O^4-alkylated-dT in SOS-induced AB1157 *E. coli* cells deficient in Pol IV. (a) Gel image showing the 13-mer and 10-mer products released from the bottom-strand (opposite to lesion-containing strand) of the PCR products of the progeny of the competitor genome and the control or lesion-carrying genome, where 10mer A, 10mer C, 10mer G, and 10mer T represent the [5′-^{32}P]-labeled standard ODNs 5′-AATTATAGCN-3′, with ‘N’ being A, C, G, and T, respectively. (b) Gel image showing the 13-mer and 10-mer products released from the top-strand (lesion-containing strand) of the PCR products of the progeny of the competitor genome and the control or lesion-carrying genome, where 10mer A, 10mer C, 10mer G, and 10mer T represent the [5′-^{32}P]-labeled standard ODNs 5′-GGCGMGCTAT-3′, with ‘M’ being A, C, G, and T, respectively.

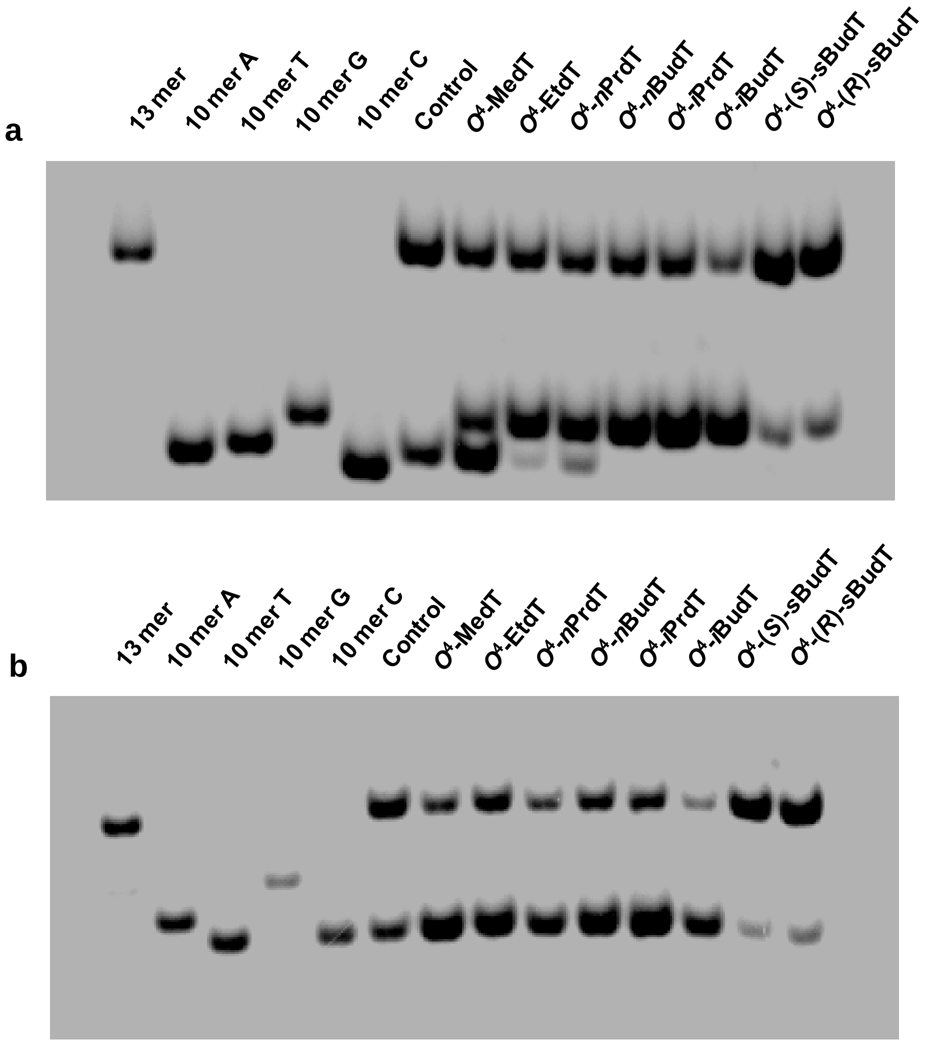

Figure S36. Native PAGE (30%) for monitoring the bypass efficiencies and mutation frequencies of O^4-alkylated-dT in SOS-induced AB1157 *E. coli* cells deficient in Pol V. (a) Gel image showing the 13-mer and 10-mer products released from the bottom-strand (opposite to lesion-containing strand) of the PCR products of the progeny of the competitor genome and the control or lesion-carrying genome, where 10mer A, 10mer C, 10mer G, and 10mer T represent the [5′-^{32}P]-labeled standard ODNs 5′-AATTATAGCN-3′, with ‘N’ being A, C, G, and T, respectively. (b) Gel image showing the 13-mer and 10-mer products released from the top-strand (lesion-containing strand) of the PCR products of the progeny of the competitor genome and the control or lesion-carrying genome, where 10mer A, 10mer C, 10mer G, and 10mer T represent the [5′-^{32}P]-labeled standard ODNs 5′-GGCGMGCTAT-3′, with ‘M’ being A, C, G, and T, respectively.

a

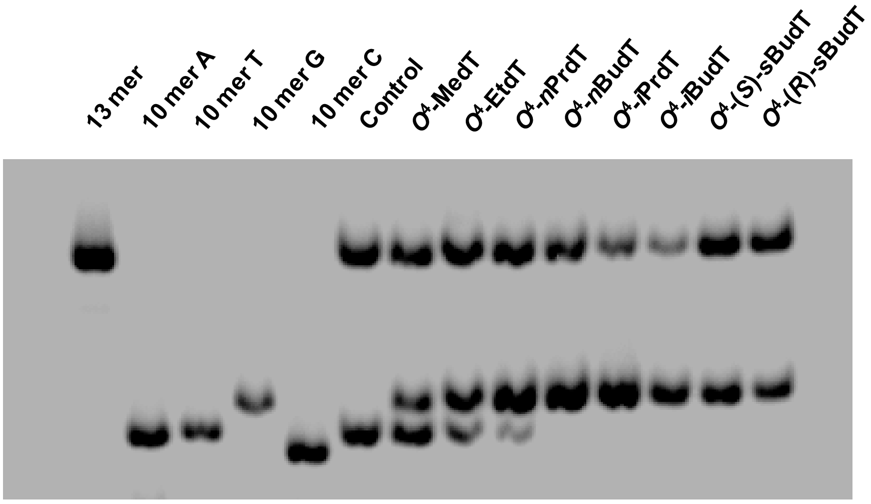

b

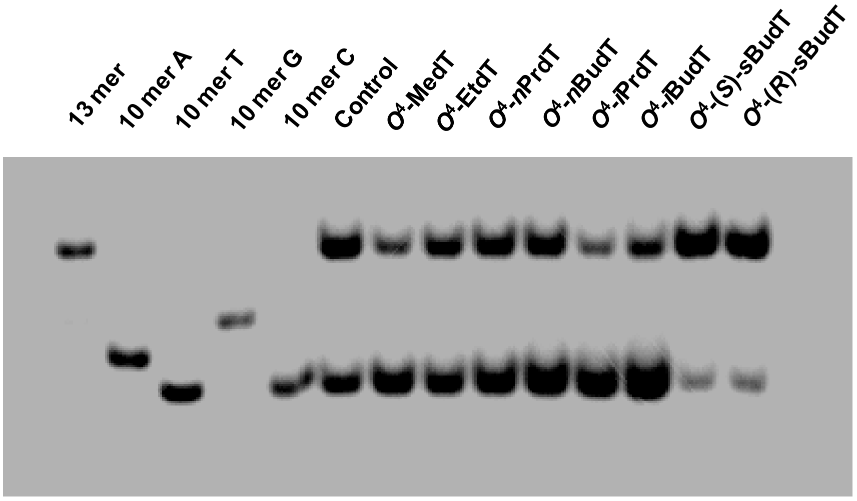

Figure S37. Native PAGE (30%) for monitoring the bypass efficiencies and mutation frequencies of O^4-alkylated-dT in SOS-induced AB1157 *E. coli* cells deficient in Pol II, Pol IV and Pol V. (a) Gel image showing the 13-mer and 10-mer products released from the bottom-strand (opposite to lesion-containing strand) of the PCR products of the progeny of the competitor genome and the control or lesion-carrying genome, where 10mer A, 10mer C, 10mer G, and 10mer T represent the [5′-^{32}P]-labeled standard ODNs 5′-AATTATAGCN-3′, with 'N' being A, C, G, and T, respectively. (b) Gel image showing the 13-mer and 10-mer products released from the top-strand (lesion-containing strand) of the PCR products of the progeny of the competitor genome and the control or lesion-carrying genome, where 10mer A, 10mer C, 10mer G, and 10mer T represent the [5′-^{32}P]-labeled standard ODNs 5′-GGCGMGCTAT-3′, with 'M' being A, C, G, and T, respectively.

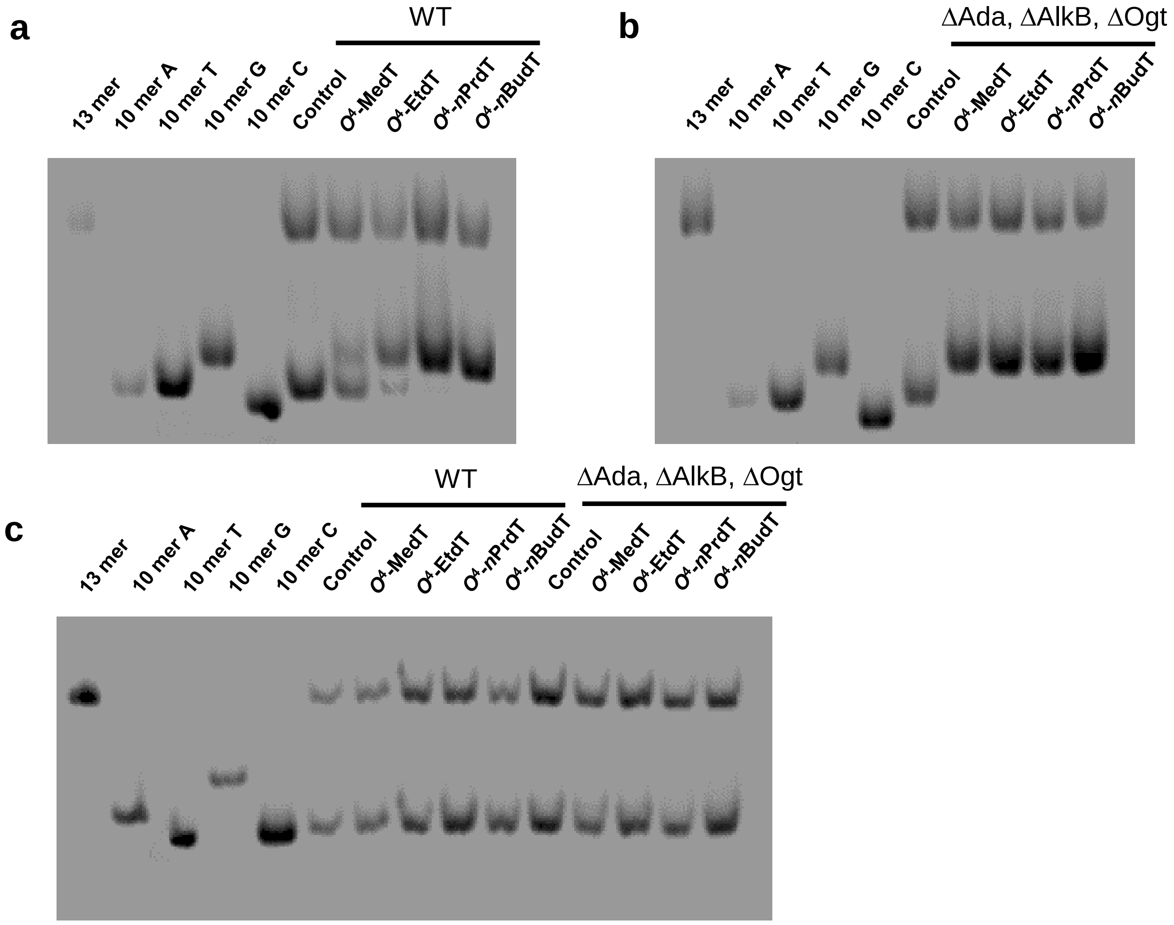

Figure S38. Native PAGE (30%) for monitoring the bypass efficiencies and mutation frequencies of O^4-alkylated-dT in SOS-induced *E. coli* cells that are proficient in O^6-alkylguanine-DNA alkyltransferases and deficient in both Ada and Ogt. (a,b) Gel image showing the 13-mer and 10-mer products released from the bottom-strand (opposite to lesion-containing strand) of the PCR products of the progeny of the competitor genome and the control or lesion-carrying genome, where 10mer A, 10mer C, 10mer G, and 10mer T represent the [5′-^{32}P]-labeled standard ODNs 5′-AATTATAGCN-3′, with ‘N’ being A, C, G, and T, respectively. (b) Gel image showing the 13-mer and 10-mer products released from the top-strand (lesion-containing strand) of the PCR products of the progeny of the competitor genome and the control or lesion-carrying genome, where 10mer A, 10mer C, 10mer G, and 10mer T represent the [5′-^{32}P]-labeled standard ODNs 5′-GGCGMGCTAT-3′, with ‘M’ being A, C, G, and T, respectively.

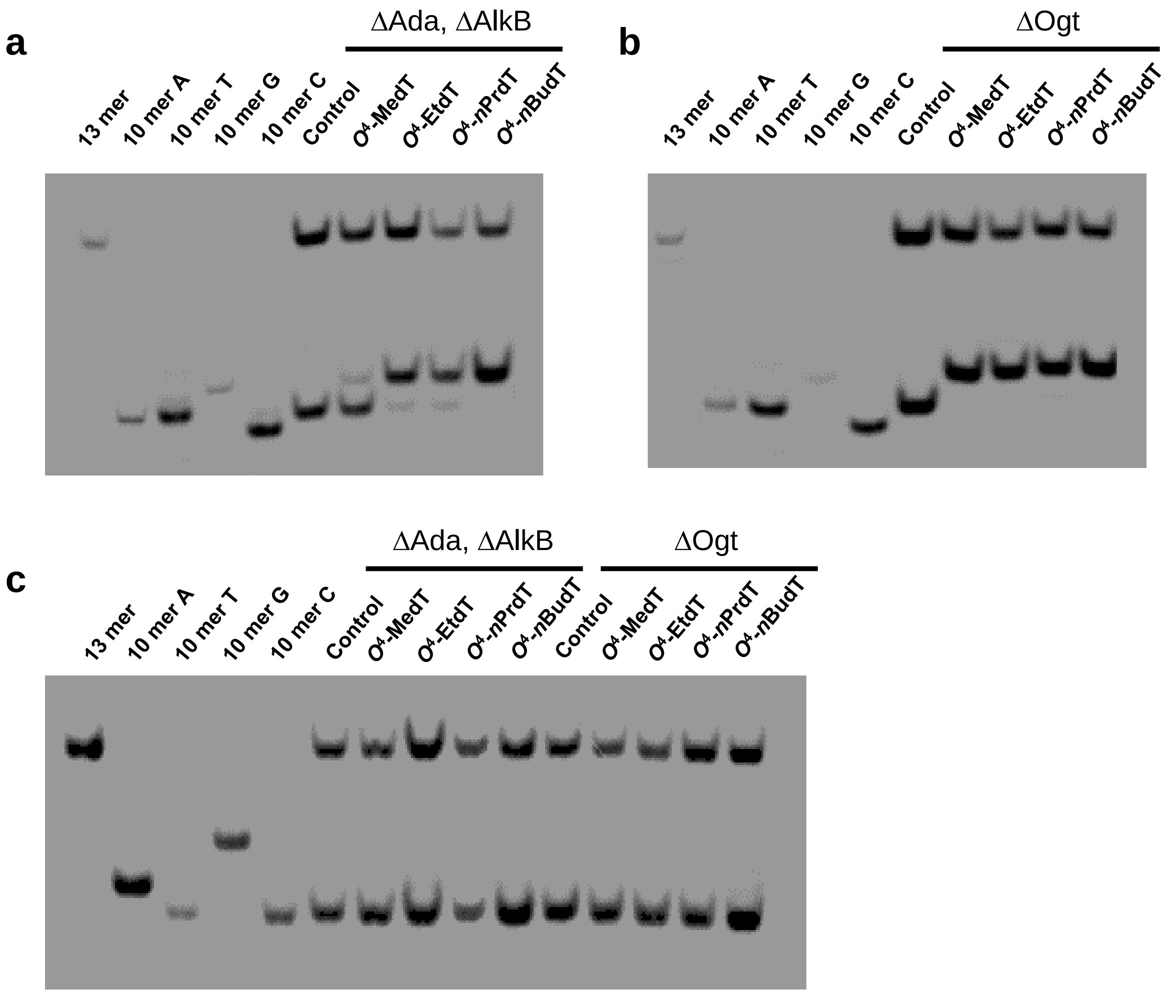

Figure S39. Native PAGE (30%) for monitoring the bypass efficiencies and mutation frequencies of *O*^{4}-alkylated-dT in SOS-induced *E. coli* cells that are deficient in *O*^{6}-alkylguanine-DNA alkyltransferase Ada or Ogt. (a,b) Gel image showing the 13-mer and 10-mer products released from the bottom-strand (opposite to lesion-containing strand) of the PCR products of the progeny of the competitor genome and the control or lesion-carrying genome, where 10mer A, 10mer C, 10mer G, and 10mer T represent the [5′-^{32}P]-labeled standard ODNs 5′-AATTATAGCN-3′, with ‘N’ being A, C, G, and T, respectively. (b) Gel image showing the 13-mer and 10-mer products released from the top-strand (lesion-containing strand) of the PCR products of the progeny of the competitor genome and the control or lesion-carrying genome, where 10mer A, 10mer C, 10mer G, and 10mer T represent the [5′-^{32}P]-labeled standard ODNs 5′-GGCGMGCTAT-3′, with ‘M’ being A, C, G, and T, respectively.
